# The growth, nutrient uptake and fruit quality in four strawberry cultivars under different Spectra of LED supplemental light

**DOI:** 10.1186/s12870-024-04880-5

**Published:** 2024-03-08

**Authors:** Hamid Reza Roosta, Mahdi Bikdeloo, Mansour Ghorbanpour

**Affiliations:** 1 Department of Horticultural Sciences, Faculty of Agriculture and Natural Resources, Arak University, Arak, 38156-8-8349 Iran; 2 Department of Medicinal Plants, Faculty of Agriculture and Natural Resources, Arak University, Arak, 38156-8-8349 Iran

**Keywords:** Blue light, Fragaria × ananassa, Greenhouse, Growth, Red light, Soilless culture, Yield

## Abstract

An experiment was conducted in a greenhouse to determine the effects of different supplemental light spectra on the growth, nutrient uptake, and fruit quality of four strawberry cultivars. The plants were grown under natural light and treated with blue (460 nm), red (660 nm), and red/blue (3:1) lights. Results showed that the “Parous” and “Camarosa” had higher fresh and dry mass of leaves, roots, and crowns compared to the “Sabrina” and “Albion”. The use of artificial LED lights improved the vegetative growth of strawberry plants. All three supplemental light spectra significantly increased the early fruit yield of cultivars except for “Parous”. The red/blue supplemental light spectrum also increased the fruit mass and length of the “Albion”. Supplemental light increased the total chlorophyll in “Camarosa” and “Albion”, as well as the total soluble solids in fruits. The “Albion” had the highest concentration of fruit anthocyanin, while the “Sabrina” had the lowest. The use of supplemental light spectra significantly increased the fruit anthocyanin concentration in all cultivars. Without supplemental light, the “Camarosa” had the lowest concentration of K and Mg, which increased to the highest concentration with the use of supplemental light spectra. All three spectra increased Fe concentration to the highest value in the “Sabrina”, while only the red/blue light spectrum was effective on the “Camarosa”. In conclusion, the use of supplemental light can increase the yield and fruit quality of strawberries by elevating nutrients, chlorophyll, and anthocyanin concentrations in plants.

## Introduction

Climate change and water scarcity have led to a decline in the availability of suitable lands for strawberry production in Iran. As a result, there has been a rise in the production of strawberries in large-scale greenhouses. However, constructing these greenhouses can be expensive, so it is crucial to use techniques that guarantee consistently high yields. Achieving this requires the development of environmental control techniques, such as light, that allow plants to attain their full photosynthetic potential. Plant growth is influenced by both genetically and environmentally factors. The response of plants to light is affected by various factors, including light quality, environmental conditions, season, genotype, and cultivation methods [1]. The quality of light significantly impacts plant growth, development and photomorphogenesis by providing effective wavelengths in photosynthesis [2]. Light is a vital source of energy for plant growth, flowering, fruiting, and photosynthesis [3]. In conditions where natural light is insufficient, supplemental lighting can effectively promote the photosynthesis and growth of strawberries. When growing strawberries in greenhouses, additional lighting is often necessary to supplement natural sunlight, which tends to be less intense during winter months. Furthermore, during the cooler winter months, day lengths are shorter, making supplemental lighting necessary to increase greenhouse crop yield.

In recent years, LED technology has emerged as the preferred supplemental lighting source due to its energy efficiency, long lifespan, and ability to produce light of specific wavelengths. LED lighting has been shown to increase overall photosynthetic pigments, promote optimal plant growth, and increase individual fruit size in strawberries. Studies suggest that a combination of blue and red wavelengths of LED lighting is usually chosen to enhance the efficiency of plant photosynthesis [4]. Moreover, Hidaka et al. [5] achieved a twofold increase in strawberry yield through the combined application of LED lighting and CO_2_ enrichment. In a previous study, it was discovered that plants grown under LED light had higher leaf photosynthesis rates [6]. LED lights consist of a combination of blue, red, and far-red spectra, which are known to promote optimal plant growth [7–9]. Blue and red lights, in particular, have been found to be beneficial when used together. For example, using blue (475 nm) LEDs along with red LEDs has resulted in larger individual strawberry fruit sizes [10]. Additionally, when grown under growth chamber conditions, strawberry plants exposed to a combination of blue and red LEDs produced higher yields and larger individual fruit sizes than those grown under red LEDs alone [11]. The utilization of red and blue wavelengths is widely acknowledged for optimizing photosynthesis [12] and improving plant quality. Red light plays a crucial role in shoot and stem elongation, phytochrome responses, and influencing changes in plant anatomy [13]. However, it is worth noting that in certain fall-flowering strawberry cultivars, red light may have a negative impact on flower bud initiation [14]. On the other hand, blue light is known to stimulate plant growth, enhance biomass production, and contribute to chlorophyll biosynthesis, stomatal opening, enzyme synthesis, phototropism, and photosynthesis [15, 16]. By harnessing both red and blue light wavelengths, it is possible to create an optimal lighting environment for plants, maximizing their growth potential while considering specific cultivar requirements and growth stages.

Different cultivars of strawberries have unique characteristics such as plant growth, fruit production rate, disease resistance, fruit ripening time, and fruit quality, such as firmness, size, color, shape, and taste. To better understand how various cultivars of strawberries grown in a greenhouse environment respond to supplemental lighting, an experiment was conducted comparing four different cultivars under the influence of LED lights with different spectra.

The study aimed to analyze the impact of these lights on plant growth and yield, including dry matter partitioning, vegetative and reproductive traits, nutrient absorption, and fruit quality. The results of this experiment will help optimize lighting strategies for different cultivars of strawberry plants, ultimately improving their functional properties.

## Materials and methods

### Plant materials and growth conditions

An experiment was conducted in 2021 at the experimental greenhouse of Arak University. Rooted strawberry plants of four different cultivars - “Sabrina”, “Albion”, “Parous”, and “Camarosa” - were obtained from a nursery in Sanandaj, Iran. These plants were then planted in a hydroponic system, in 4-L pots that contained a mix of cocopeat and perlite in the ratio of 70:30 V: V. Three pots were assigned to each treatment, and three plants were planted in each pot. The plants were irrigated twice a day, at 10 a.m. and 3 p.m. using a drip system and a pump. During each irrigation, 150 ml of nutrient solution was given to the plants. The plants were grown in a greenhouse, with a temperature that ranged between 16 and 24 ºC, a photoperiod of 11/13 h (light/dark), a relative humidity (RH) of 50 ± 10%, and a maximum light intensity of 925 µmol m^− 2^ s^− 1^ (LED + ambient light) per day above the canopy. The plants were watered with Morgan nutrient solution [(pH: 6.5; electrical conductivity (EC): 1.4 dS.m^− 1^)], and were treated with four different light spectrums [17] Table 1.

**Table 1:** Concentration of nutrients used in the nutrient solution of this experiment

Macronutrients	Concentration (mmol.L^-1^)	Micronutrients	Concentration (µmol.L^-1^)
N	9.14	Fe	16.18
P	1.87	Mn	11.83
K	5.40	Zn	0.87
Ca	2.60	B	11.32
Mg	1.65	Cu	0.28
S	1.68	Mo	0.58

### LED tubes and light treatments

**Fig. 1:**
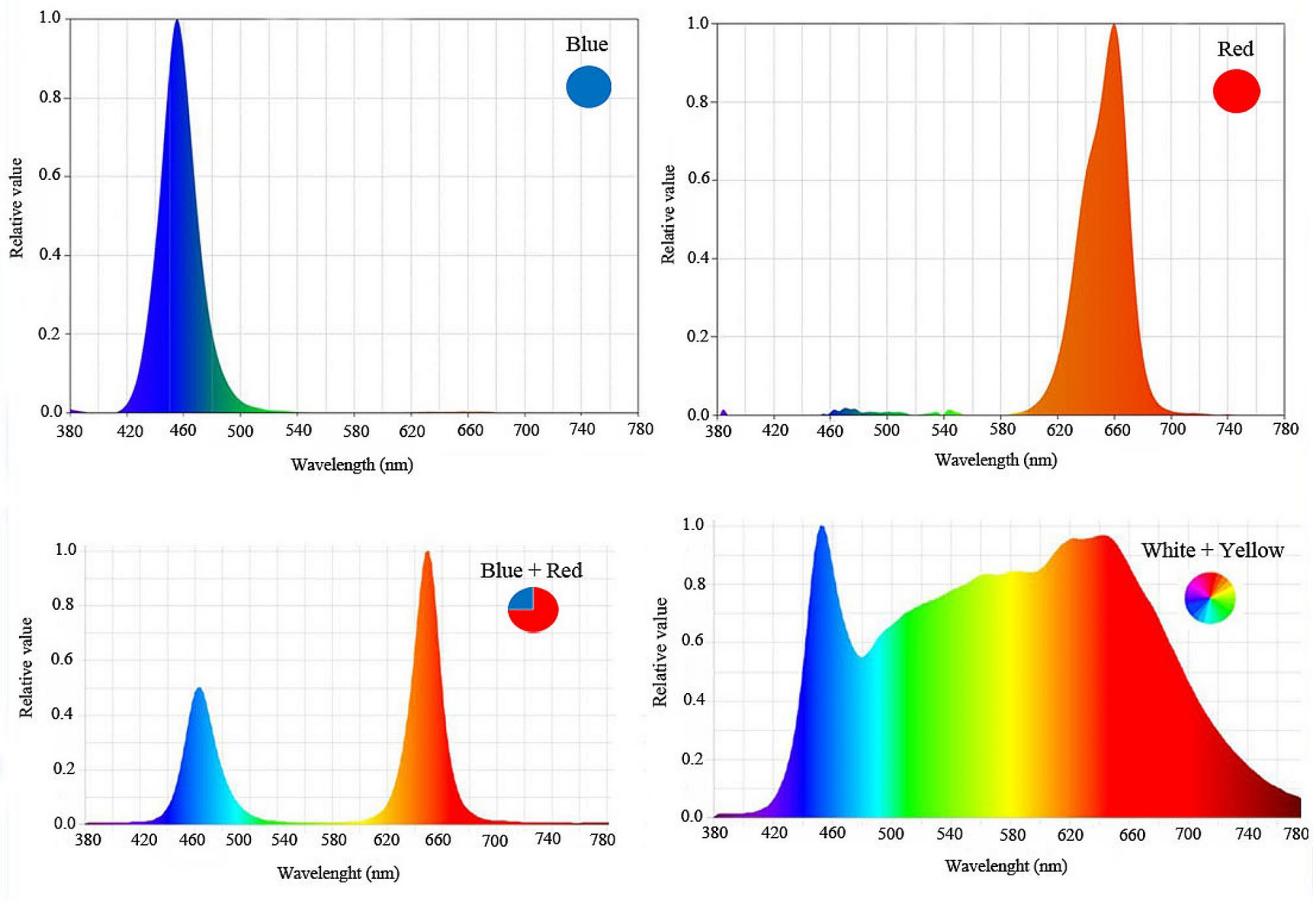
Relative distribution of different spectral LEDs (monochromatic blue, monochromatic red, red/blue (1:3) and white/ yellow (1:1) used during plant growth

The plants were grown in a greenhouse under metal structures, using LED tubes with a power of 24 watts and a photon flux density (PPFD) of 200 µmol m^− 2^ s^− 1^, as a supplemental light. The LED tubes had different spectral ranges, including monochromatic blue (B) with a peak of 460 nm, monochromatic red (R) with a peak of 660 nm, dichromatic red/blue (3:1) as shown in Fig. 1, and ambient light only according to Table 2. The LED lighting systems were mounted 30 cm apart, and the plants were grown with a photoperiod of 11 h light and 13 h dark at the leaf surface. The plants received a total photon flux density of 925 µmol m^− 2^ s^− 1^, which was a combination of both LED and ambient light.

**Table 2:** Characteristics of LEDs used in this experiment

Manufacture company	CRI	Number of LEDs	Light coverage area	Power consumption	Lens type	Certificate
Iran Grow Light	95%	6	40 cm×100 cm	24 watts	90°	CCC, CE, FEC, Ip45, RoHS

### Vegetative parameters

At the end of the experiment, the plants were collected and separated into their leaves, roots, and crowns. The samples were then weighed to record their fresh mass. To determine their dry mass, the samples were placed in an oven at 70 °C for 72 h, and their dry mass was recorded. Additionally, the length of the roots was measured using a ruler.

### Reproductive characteristics

During the growth period, various measurements were taken including early yield, fruit number, fruit length, single fruit mass, and the number of inflorescences and flowers.

### Leaf pigments and leaf gas exchange

To determine the levels of chlorophyll and carotenoids in a leaf sample, 0.25 g of frozen leaves were crushed using liquid nitrogen in a Chinese mortar. Subsequently, 20 ml of 80% acetone was added to the sample and then centrifuged at 4800 rpm for 20 min. The sample’s luminescent absorption was noted at 470, 647, and 663 nm using a spectrophotometer. Finally, the total amount of chlorophyll and carotenoids was calculated by Lichtenthaler’s method [18].

Gas exchange parameters of plants were measured using a portable photosynthesis system provided by ADC BioScientific Ltd, Hoddesdon, UK. These parameters include the net photosynthetic CO_2_ assimilation rate (*A*, µmol CO_2_ m^− 2^ s^− 1^) and stomatal conductance (*gs*, mol H_2_O m^− 2^ s^− 1^). The measurements were taken on completely expanded leaves at around 9:00 AM and 12:00 PM, 60 days after planting.

### Fruit quality

A refractometer (PAL-1, Atago Co., Ltd; Japan) was used to measure the Total Soluble Solids (°Brix) of fruits. Method developed by Nogues and Baker [19] was used to determine the content of anthocyanin. One g of fresh leaf tissue was homogenized in 10 mL of acidic methanol and then was centrifuged at 3500 rpm. The samples were measured for absorbance at 530 and 657 nm. To determine the titratable acidity of the fruit extract, 0.01 N NaOH was titrated to the endpoint of pH 8.1. The results were expressed as mg equivalents of citric acid per 100 g of fresh mass [20].

### Elemental analysis

To determine the concentrations of potassium, magnesium, and iron in plant shoots, standard methods were followed after digesting the samples using HNO_3_. First, each sample were weighed and powdered. Then, approximately 0.3 g of dry and powdered leaf samples, HNO_3_ (6 mL), and H_2_O_2_ (2 mL) were mixed in a beaker vessel. The hot plate method were used with a watch glass placed on top of the vessel to digest the samples. Hydrogen peroxide was added to help digest the organic matrix, as recommended in Thermo Scientific AN 443,662 and AN 4,344,620. A hot plate digestion system were used with a stirrer and temperature sensor to heat the samples. The samples were heated according to the following temperature program: 50 °C for 1 h, 150 °C for 1 h, and then 200 °C for another hour until brown gas was produced. The samples were left for 15 min to remove any remaining gas. After digestion, each sample were transferred to a volumetric flask (25 mL) and made up the volume with ultrapure water before analyzing the samples by ICP-OES (Analytic Jena Co., Germany). The nutrient analysis results were converted from concentration units to nutrient content per total plant content by multiplying the nutrient concentration in the plant organs by the dry mass accumulation of the entire plant.

### Experimental design and data analysis

The study was conducted using a completely randomized design (CRD) with two factors (supplemental light spectra as fixed factor and cultivar as random factor) in three replications (*n* = 3), as a factorial experiment, with three single plants in pots. The data was analyzed using SAS software (SAS Institute, Cary, NC, USA), employing a two-way ANOVA model. In case of significant treatment effects in the ANOVA, the multiple ranges Duncan test was used as a post hoc to calculate significant mean differences (*P* < 0.05). After demonstrating the differences between the means, post hoc range tests and pairwise multiple comparisons were employed to determine which means were different. The graphs were created using Excel 2016.

## Results

### Vegetative characteristics

The experiment results indicate that the “Parous” and “Camarosa” had a higher fresh and dry mass of leaves, roots, and crowns compared to the “Sabrina” and “Albion” (Table 3). “Parous” exhibited the highest leaf mass, but the different spectrums of supplemental light had no significant effect on the leaf mass of the strawberry cultivars. The dry mass of leaves in the “Parous” was significantly increased with blue light treatment compared to the control, while the root mass was increased with red/blue light treatment in “Sabrina” and “Albion” compared to the control. “Sabrina” increased the root fresh mass with the blue light treatment and “parous” with the red light treatment. However, root dry mass increased only in the “Camarosa” under the influence of red light compared to the control. “Sabrina” increased the crown fresh mass with the red light treatment and “Camarosa” with the blue light treatment, while crown dry mass only increased in “Parous” and “Camarosa” by the combination of red/blue light (Table 3). “Parous” cultivar exhibited the highest crown diameter among the cultivars. The red/blue spectrum of supplemental light increased the crown diameter in the “Camarosa”, while other spectra had no significant effect on it (Table 3). “Parous” and “Camarosa” had the longest roots, and different light spectrums did not affect root length (Table 3). “Parous” also had the highest number of leaves among the cultivars, and the blue spectrum of LED increased the number of leaves in this cultivar. Whereas, in the “Camarosa”, it was the red spectrum of light that increased the number of leaves (Table 3).

**Table 3:** Effect of different supplemental LED light spectra (red/blue, 3:1; R: B, with a peak 656 nm, blue, with a peak at 450 nm, red, with a peak at 656 nm, control (ambient light)) on vegetative growth characteristics (LFM: leaf fresh mass, CFM: crown fresh mass, RFM: root fresh mass, LDM: leaf dry mass, CDM: crown dry mass, RDM: root dry mass, CD: crown diameter, RL: root length, LN: leaf number) of four strawberry cultivars. The photosynthetic photon flux density (PPFD) was 215 ± 5 mol m− 2 s− 1. The photoperiod of 11/13 h (day/ night) was maintained

Strawberry cultivar	Light spectrum	LFM (g plant^− 1^)	CFM (g plant^− 1^)	RFM (g plant^− 1^)	LDM (g plant^− 1^)	CDM (g plant^− 1^)	RDM (g plant^− 1^)	CD (mm)	RL (cm)	LN (plant^− 1^)
“Sabrina”	Control	12.32 ± 0.42hi	3.08 ± 0.30 h	3.42 ± 0.24 g	5.03 ± 0.58 fg	0.55 ± 0.02 h	2.96 ± 0.40d	10.52 ± 0.27de	14.00 ± 0.76 g	67 ± 10.5def
	Red	10.81 ± 0.60i	5.28 ± 0.51c-g	3.67 ± 0.13 g	4.94 ± 0.50 g	0.72 ± 0.07gh	3.27 ± 0.05d	12.44 ± 0.41a-e	15.42 ± 0.58 fg	66 ± 3.6def
	Blue	16.45 ± 1.11f-i	3.95 ± 0.77fgh	7.53 ± 0.50f	8.79 ± 0.87c-f	0.81 ± 0.10fgh	3.69 ± 0.29d	11.54 ± 0.85b-e	14.83 ± 0.83 fg	77 ± 7.2cde
	Red/Blue	14.15 ± 1.77ghi	3.41 ± 0.38gh	8.15 ± 0.11f	7.09 ± 1.48efg	0.68 ± 0.07gh	4.61 ± 0.27 cd	10.28 ± 0.75e	16.33 ± 0.17efg	67 ± 10.4def
“Albion”	Control	18.69 ± 0.79 fg	3.89 ± 0.44fgh	9.54 ± 0.73f	6.95 ± 0.20efg	0.83 ± 0.07fgh	4.64 ± 0.50 cd	11.59 ± 0.88b-e	16.67 ± 1.01d-g	69 ± 4.6de
	Red	17.13 ± 0.38fgh	4.24 ± 0.30e-h	7.54 ± 0.58f	6.47 ± 0.58 fg	0.78 ± 0.06fgh	3.42 ± 0.25d	9.95 ± 0.38e	15.42 ± 0.71 fg	61 ± 4.4ef
	Blue	17.37 ± 0.09fgh	5.66 ± 0.44c-f	10.23 ± 0.13ef	7.63 ± 0.58d-g	1.01 ± 0.09efg	5.05 ± 0.41 cd	11.94 ± 0.78a-e	17.17 ± 0.67c-f	77 ± 12.5cde
	Red/Blue	20.33 ± 1.51f	5.9 ± 0.10b-f	16.82 ± 0.93bc	8.79 ± 0.75c-f	1.17 ± 0.8def	6.87 ± 1.18bc	13.03 ± 0.73a-d	19.50 ± 0.29a-d	65 ± 7.8ef
“Parous”	Control	40.77 ± 1.11ab	6.12 ± 0.53b-e	13.64 ± 1.29de	10.39 ± 0.78bcd	1.01 ± 0.12efg	6.79 ± 0.46bc	14.31 ± 1.15a	21.67 ± 0.88ab	104 ± 11.1b
	Red	40.52 ± 2.65ab	6.70 ± 0.54a-d	18.86 ± 3.04a	11.24 ± 3.16a-d	1.23 ± 0.09cde	8.75 ± 1.56ab	14.10 ± 0.65ab	21.17 ± 1.45ab	88 ± 8.2bcd
	Blue	45.94 ± 4.5a	6.67 ± 0.58a-d	15.88 ± 0.22 cd	14.33 ± 1.33a	1.31 ± 0.13cde	6.84 ± 0.20bc	13.79 ± 0.84abc	20.00 ± 0.76abc	134 ± 17.4a
	Red/Blue	40.27 ± 1.98bc	6.80 ± 0.89a-c	16.37 ± 1.84bcd	12.58 ± 1.27abc	1.42 ± 0.17bcd	6.70 ± 1.29bc	13.52 ± 1.07abc	19.00 ± 1.04b-e	96 ± 9.6bc
“Camarosa”	Control	26.64 ± 2.18e	6.21 ± 0.56b-e	13.21 ± 0.74de	9.51 ± 1.36cde	1.34 ± 0.08cde	5.60 ± 0.26 cd	11.18 ± 0.70cde	21.50 ± 1.04ab	77 ± 9.5cde
	Red	27.48 ± 1.82de	7.23 ± 0.03abc	15.34 ± 0.46 cd	12.37 ± 0.19abc	1.57 ± 0.21bc	8.77 ± 1.45ab	12.22 ± 0.22a-e	21.33 ± 0.93ab	100 ± 20.2b
	Blue	28.24 ± 1.70 cd	8.39 ± 0.19a	13.60 ± 0.56 cd	9.61 ± 0.65cde	1.75 ± 0.24ab	6.45 ± 1.11bc	13.19 ± 1.32abc	20.67 ± 0.44abc	89 ± 14.8bc
	Red/Blue	27.92 ± 2.12de	7.88 ± 1.31ab	15.49 ± 0.59 cd	10.11 ± 0.32b-e	2.06 ± 0.11a	5.39 ± 0.25 cd	14.18 ± 0.59ab	22.00 ± 1.61a	91 ± 23.6bc

Mean separation was done by Duncan’s multiple range test and the same letter(s) in each column indicates non-significant difference at *P* ≤ 0.05

### Reproductive characteristics

The “Parous” had the highest number of early fruits, and the application of the red/blue supplemental light spectrum significantly increased the number of early fruits in all tested cultivars compared to the control (Table 4). However, in the “Sabrina”, blue and red light spectrums alone and in the “Camarosa” blue light spectrum alone also increased the number of early fruits. The highest early ripe fruit also belonged to the “Parous”, but the light spectrum did not affect it (Table 4). Although the number of early ripe fruits in the “Sabrina” was affected by blue and red/blue light, the “Albion” was significantly increased under the influence of the red/blue light spectrum, and in the “Camarosa” was significantly increased under the influence of blue spectrum. The “Parous” had the highest early fruit yield, which was not affected by any of the supplemental light spectrums (Table 4). However, in the other three cultivars, all three tested supplemental light spectrums caused a significant increase in early fruit yield, so that this trait reached the highest level in the “Albion” treated with red/blue light spectrum. In the absence of supplemental light, the number of the inflorescence of cultivars did not differ significantly, but with the use of supplemental light spectrums of sole red and blue in the “Sabrina”, blue alone and red/blue in the “Albion”, and red/blue in the “Camarosa”, the number of the inflorescence significantly increased (Table 4). The “Parous” had the highest number of flowers, and the number of flowers significantly increased under the influence of blue light in the “Sabrina”, “Albion”, and “Parous” (Table 4). The spectrum of red/blue light played a positive role in the number of flowers of the “Albion”. In the absence of supplemental light, the fruit mass and fruit length of the cultivars did not differ significantly, but with the use of the red/blue supplemental light spectrum, fruit mass and length increased significantly in the “Albion” (Table 4). Fruit mass and length of other cultivars were not affected by different light spectrums.

**Table 4:** Effect of different supplementary LED light spectra (red/blue, 3:1; R: B, with a peak 656 nm, blue, with a peak at 450 nm, red, with a peak at 656 nm, control (ambient light)) on reproductive growth characteristics of four strawberry cultivars. The photosynthetic photon flux density (PPFD) was 215 ± 5 mol m− 2 s− 1. The photoperiod of 11/13 h (day/ night) was maintained

Strawberry cultivar	Light spectrum	Fruit No. (plant^− 1^)	Inflorescence No. (plant^− 1^)	Flower No. (plant^− 1^)	Ripe fruit No. (plant^− 1^)	Fruit length (mm)	Fruit mass (g fruit^− 1^)	Early fruit yield (g plant^− 1^)
“Sabrina”	Control	1.33 ± 0.58d	2.33 ± 1.15c	4.67 ± 2.91 fg	1.33 ± 0.58bc	25.21 ± 8.54 cd	4.39 ± 1.63e	5.22 ± 3.39ij
	Red	6.00 ± 2.67abc	4.33 ± 1.45b	5.00 ± 1.86 fg	3.33 ± 1.45abc	24.56 ± 1.16 cd	5.78 ± 0.50cde	17.34 ± 1.49f
	Blue	5.33 ± 3.18bc	5.00 ± 2.51b	22.00 ± 8.32b	5.00 ± 2.33a	26.04 ± 2.62bcd	7.51 ± 1.48b-e	18.85 ± 6.25ef
	Red/Blue	5.00 ± 2.00bc	2.67 ± 3.00c	3.00 ± 3.00 g	5.00 ± 1.33a	28.93 ± 9.84bcd	4.17 ± 2.12e	19.59 ± 6.17ef
“Albion”	Control	1.67 ± 0.67d	2.67 ± 1.20c	2.67 ± 1.97 g	1.00 ± 0.33c	25.00 ± 12.33 cd	7.58 ± 4.92b-e	5.25 ± 4.92ij
	Red	3.67 ± 2.03 cd	3.67 ± 2.03bc	6.00 ± 3.18efg	2.33 ± 1.15abc	25.35 ± 2.06 cd	5.41 ± 1.21de	10.62 ± 6.42gh
	Blue	3.67 ± 1.67 cd	4.33 ± 1.86b	15.33 ± 9.87 cd	2.33 ± 0.67abc	22.87 ± 8.01d	5.88 ± 2.03cde	10.58 ± 2.03gh
	Red/Blue	9.00 ± 6.02a	9.66 ± 4.63a	18.33 ± 8.33bc	5.00 ± 3.67a	40.31 ± 0.93a	14.93 ± 0.76a	49.39 ± 3.33a
“Parous”	Control	5.67 ± 3.48 cd	4.00 ± 2.31bc	9.67 ± 6.12ef	3.33 ± 1.76abc	31.45 ± 1.73bc	8.78 ± 0.62b-e	26.34 ± 1.86 cd
	Red	5.67 ± 1.76bc	5.33 ± 1.33b	11.00 ± 15.76de	3.33 ± 1.33abc	26.25 ± 3.80bcd	8.52 ± 3.27b-e	23.38 ± 10.68cde
	Blue	6.67 ± 3.33abc	5.67 ± 2.96b	27.00 ± 12.00a	3.33 ± 1.15abc	33.11 ± 0.94b	11.42 ± 0.76ab	22.53 ± 10.99de
	Red/Blue	9.00 ± 6.24a	3.67 ± 2.33bc	9.33 ± 7.55ef	4.33 ± 2.96abc	30.56 ± 0.34bc	10.28 ± 1.03bc	27.88 ± 5.53c
“Camarosa”	Control	1.33 ± 0.88d	2.00 ± 1.15c	6.00 ± 4.16efg	1.00 ± 0.33c	24.46 ± 7.91 cd	5.18 ± 1.80de	3.85 ± 1.80j
	Red	4.33 ± 2.19 cd	2.00 ± 1.00c	4.67 ± 3.70 fg	3.00 ± 1.53abc	25.04 ± 1.75 cd	5.22 ± 0.81de	14.99 ± 2.23 fg
	Blue	8.33 ± 6.03ab	4.00b ± 2.61c	10.33 ± 8.43e	4.67 ± 3.23ab	31.75 ± 1.04bc	9.79 ± 1.94bcd	39.18 ± 7.77b
	Red/Blue	6.33 ± 4.41abc	5.00 ± 3.60b	7.67 ± 5.45efg	4.00 ± 3.06abc	30.36 ± 1.99bcd	8.73 ± 0.71b-e	34.92 ± 2.84b

Mean separation was done by Duncan’s multiple range test and the same letter(s) in each column indicates non-significant difference at *P* ≤ 0.05.

### Leaf pigments, photosynthesis rate and stomatal conductance

**Fig. 2:**
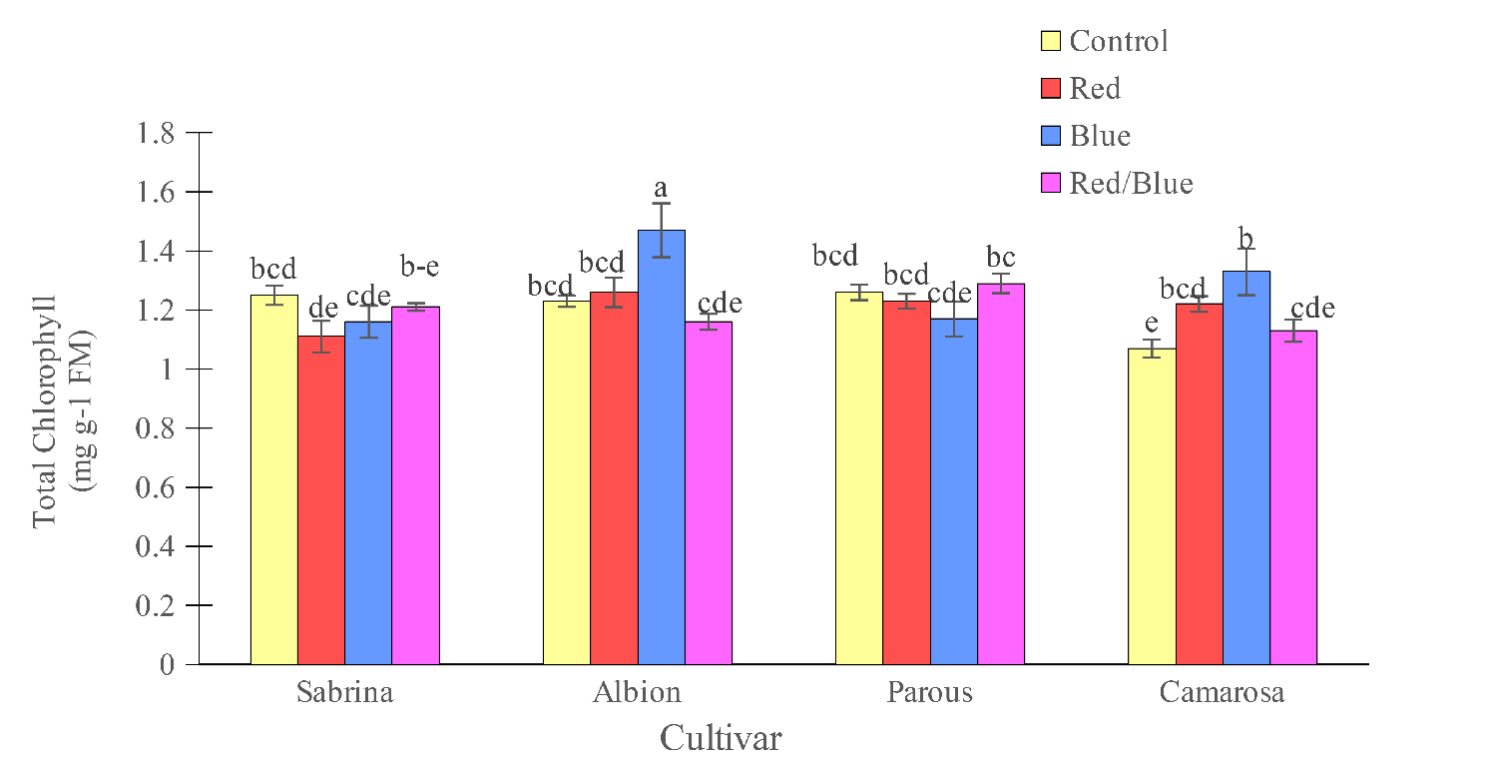
Effect of different supplemental LED light spectra (red/blue, 3:1; R: B, with a peak of 656 nm, blue, with a peak at 450 nm, red, with a peak at 656 nm, control (ambient light)) on leaf total chlorophyll concentration of four strawberry cultivars. The photosynthetic photon flux density (PPFD) was 215 ± 5 µmol m^− 2^ s^− 1^. The photoperiod of 11/13 h (day/ night) was maintained. Bars with different letters show significant differences at *P* ≤ 0.05 (Duncan)

**Fig. 3:**
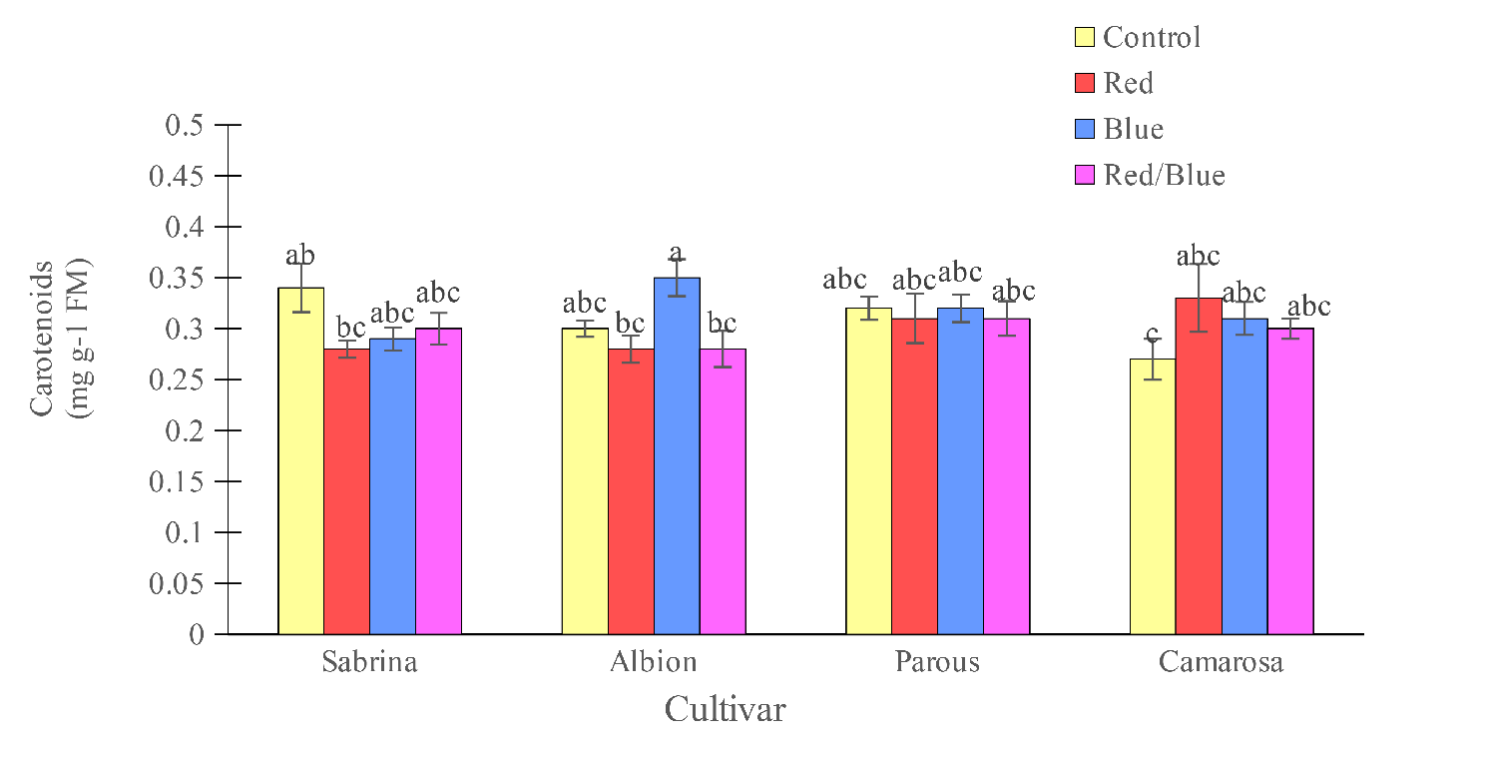
Effect of different supplemental LED light spectra (red/blue, 3:1; R: B, with a peak of 656 nm, blue, with a peak at 450 nm, red, with a peak at 656 nm, control (ambient light)) on leaf carotenoids concentration of four strawberry cultivars. The photosynthetic photon flux density (PPFD) was 215 ± 5 µmol m^− 2^ s^− 1^. The photoperiod of 11/13 h (day/ night) was maintained. Bars with different letters show significant differences at *P* ≤ 0.05 (Duncan)

Figures 2 and 3 reveal that the “Camarosa” had the lowest chlorophyll and total carotenoids in the absence of supplemental light. “Albion” increased the chlorophyll content with the blue light treatment and “Camarosa” with the red and blue treatments. It should be noted that the different light spectrums did not have any effect on the concentration of carotenoids in the leaves.

**Fig. 4:**
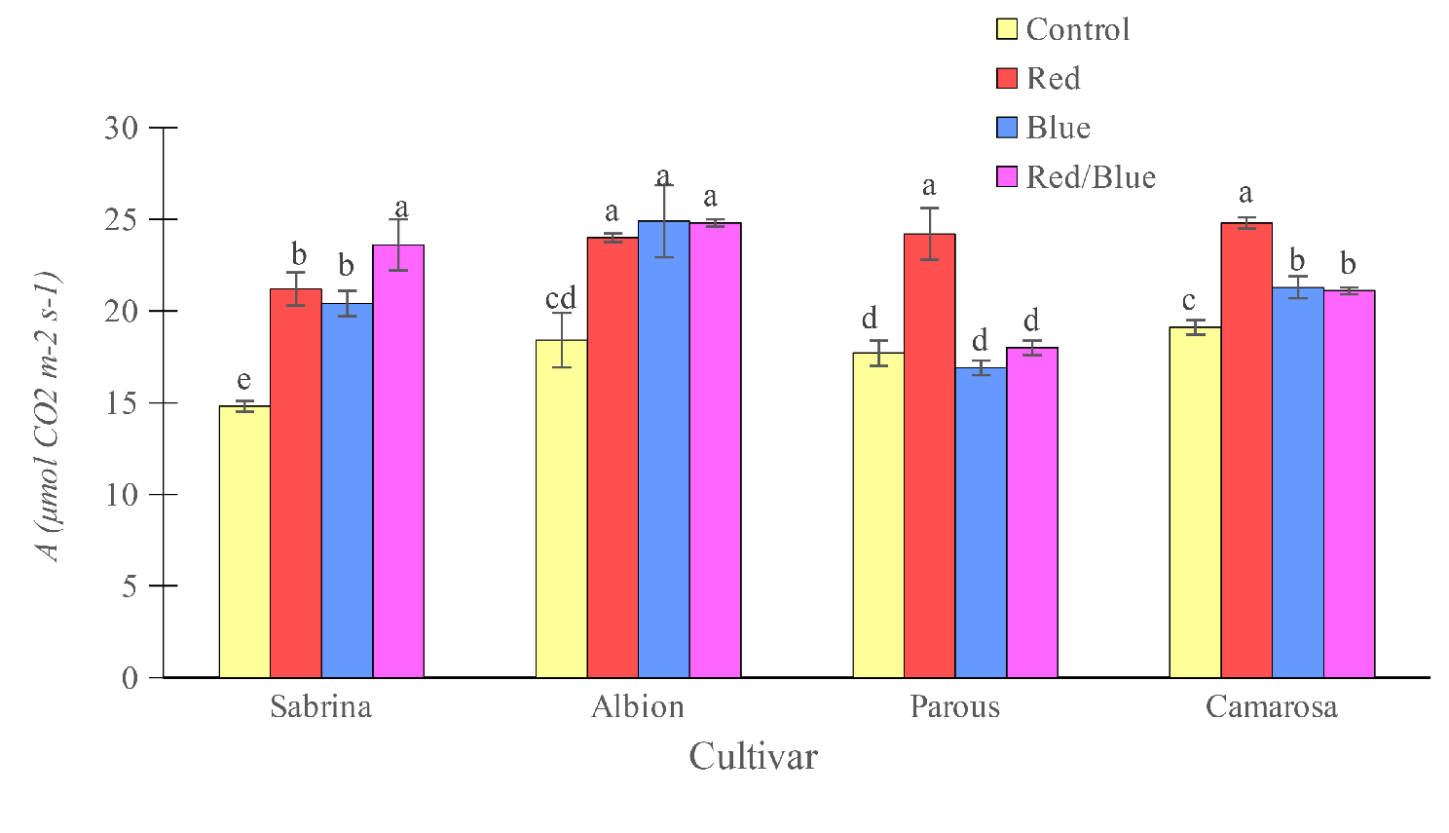
Effect of different supplemental LED light spectra (red/blue, 3:1; R: B, with a peak of 656 nm, blue, with a peak at 450 nm, red, with a peak at 656 nm, control (ambient light)) on net photosynthetic CO_2_ assimilation rate of four strawberry cultivars. The photosynthetic photon flux density (PPFD) was 215 ± 5 µmol m^− 2^ s^− 1^. The photoperiod of 11/13 h (day/ night) was maintained. Bars with different letters show significant differences at *P* ≤ 0.05 (Duncan)

**Fig. 5:**
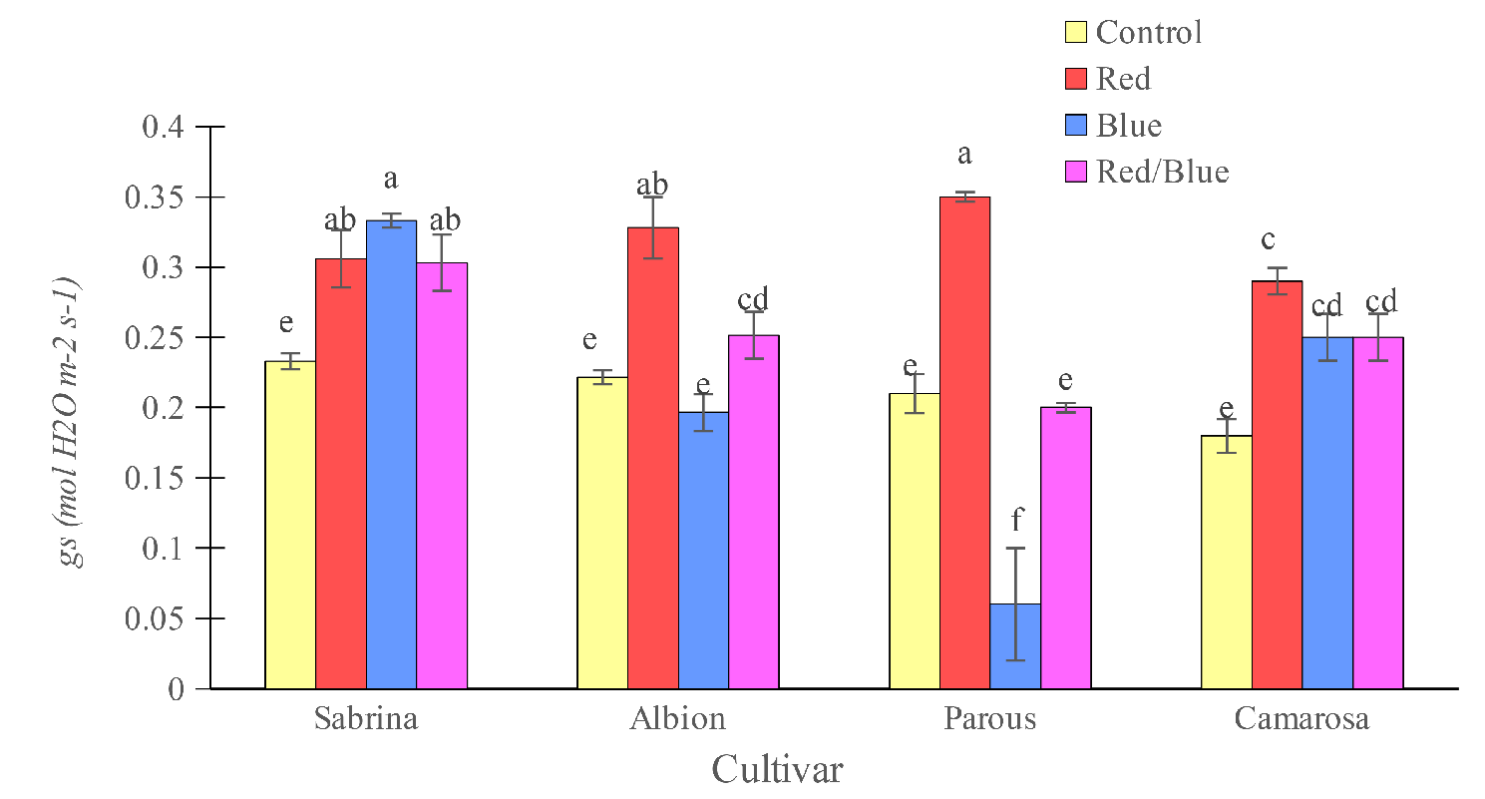
Effect of different supplemental LED light spectra (red/blue, 3:1; R: B, with a peak of 656 nm, blue, with a peak at 450 nm, red, with a peak at 656 nm, control (ambient light)) on leaf stomatal conductance of four strawberry cultivars. The photosynthetic photon flux density (PPFD) was 215 ± 5 µmol m^− 2^ s^− 1^. The photoperiod of 11/13 h (day/ night) was maintained. Bars with different letters show significant differences at *P* ≤ 0.05 (Duncan)

The rate of net photosynthetic CO_2_ assimilation (*A*) was found to increase with all supplemental light spectra in the “Sabrina,” “Albion,” and “Camarosa” when compared to control plants (Fig. 4). In “Parous,” only red light positively affected this parameter. While there was no difference among supplemental light treatments in “Albion,” in “Parous” and “Camarosa,” red light caused the highest *A*. On the other hand, in “Sabrina,” red/blue light resulted in the highest *A* in plants. Stomatal conductance (*gs*) increased in all supplemental light spectra treatments for “Sabrina” and “Camarosa” compared to the control plants (Fig. 5). In “Parous”, only the red light had a positive effect on this parameter. Although there was no difference among the supplemental light treatments for “Sabrina” and “Camarosa”, “Albion” and “Parous” showed the highest *gs* with red light, while in “Parous”, plants displayed a reduction in *gs* with blue light.

### Fruit quality

**Fig. 6:**
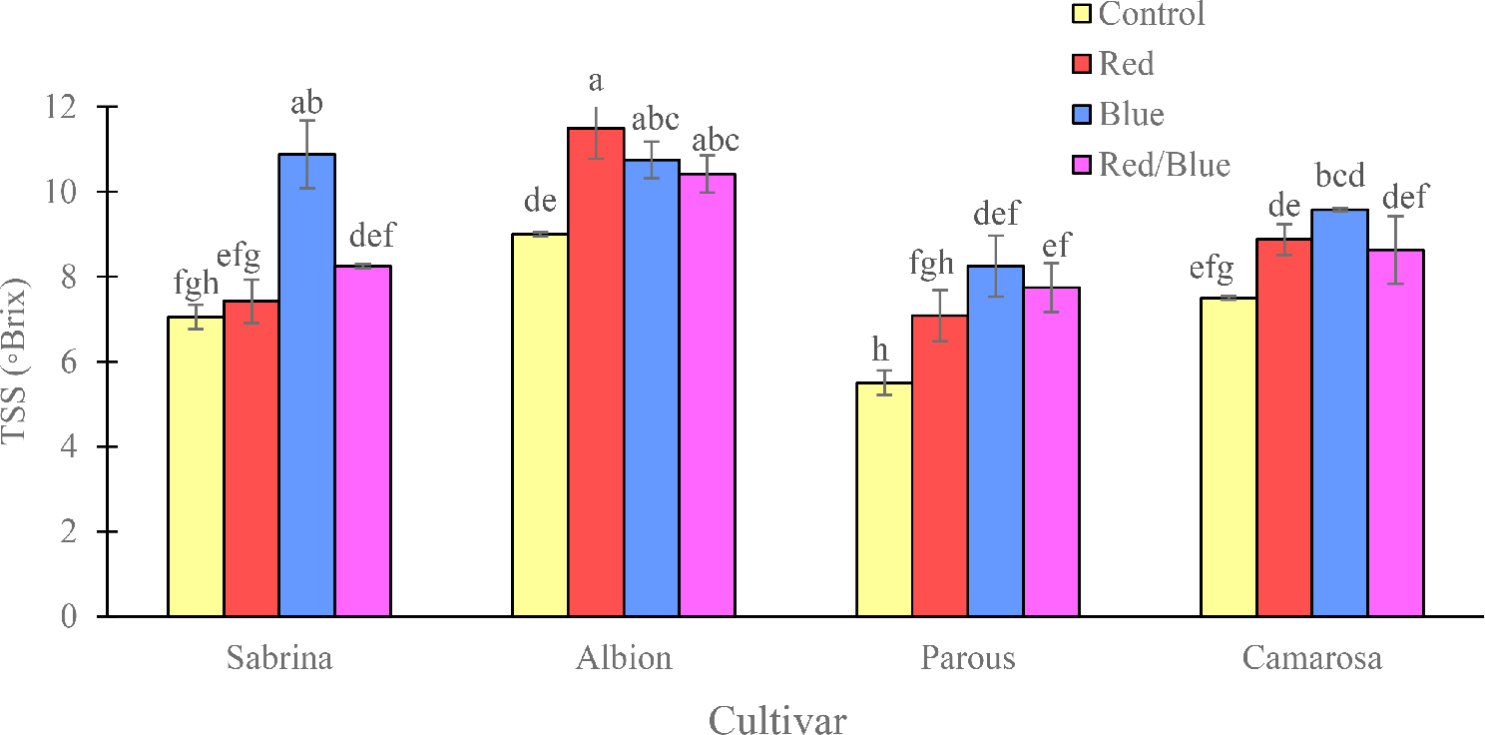
Effect of different supplemental LED light spectra (red/blue, 3:1; R: B, with a peak of 656 nm, blue, with a peak at 450 nm, red, with a peak at 656 nm, control (ambient light)) on total soluble solids of four strawberry cultivars. The photosynthetic photon flux density (PPFD) was 215 ± 5 µmol m^− 2^ s^− 1^. The photoperiod of 11/13 h (day/ night) was maintained. Bars with different letters show significant differences at *P* ≤ 0.05 (Duncan)

**Fig. 7:**
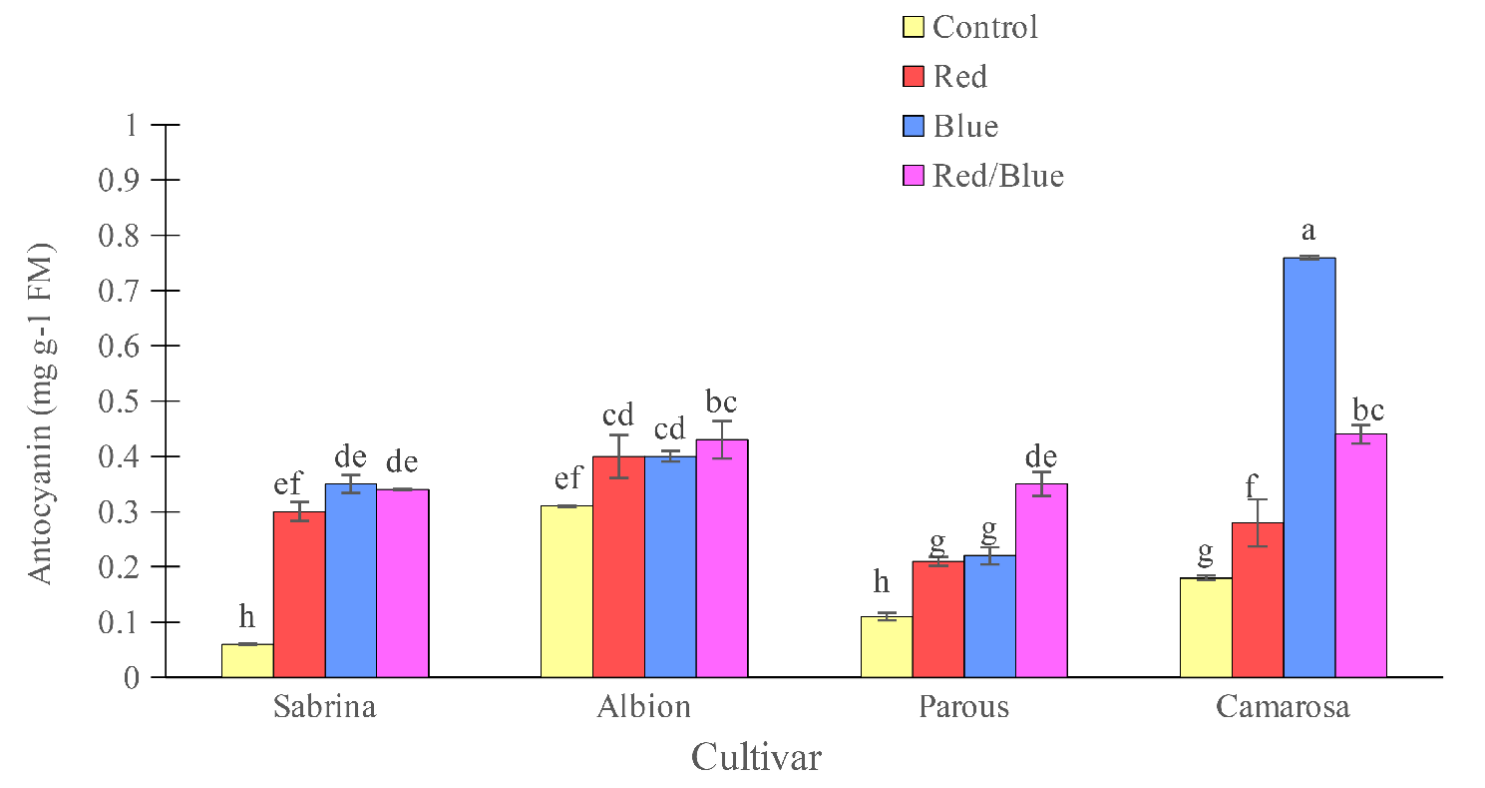
Effect of different supplemental LED light spectra (red/blue, 3:1; R: B, with a peak of 656 nm, blue, with a peak at 450 nm, red, with a peak at 656 nm, control (ambient light)) on anthocyanin of four strawberry cultivars. The photosynthetic photon flux density (PPFD) was 215 ± 5 µmol m^− 2^ s^− 1^. The photoperiod of 11/13 h (day/ night) was maintained. Bars with different letters show significant differences at *P* ≤ 0.05 (Duncan)

**Fig. 8:**
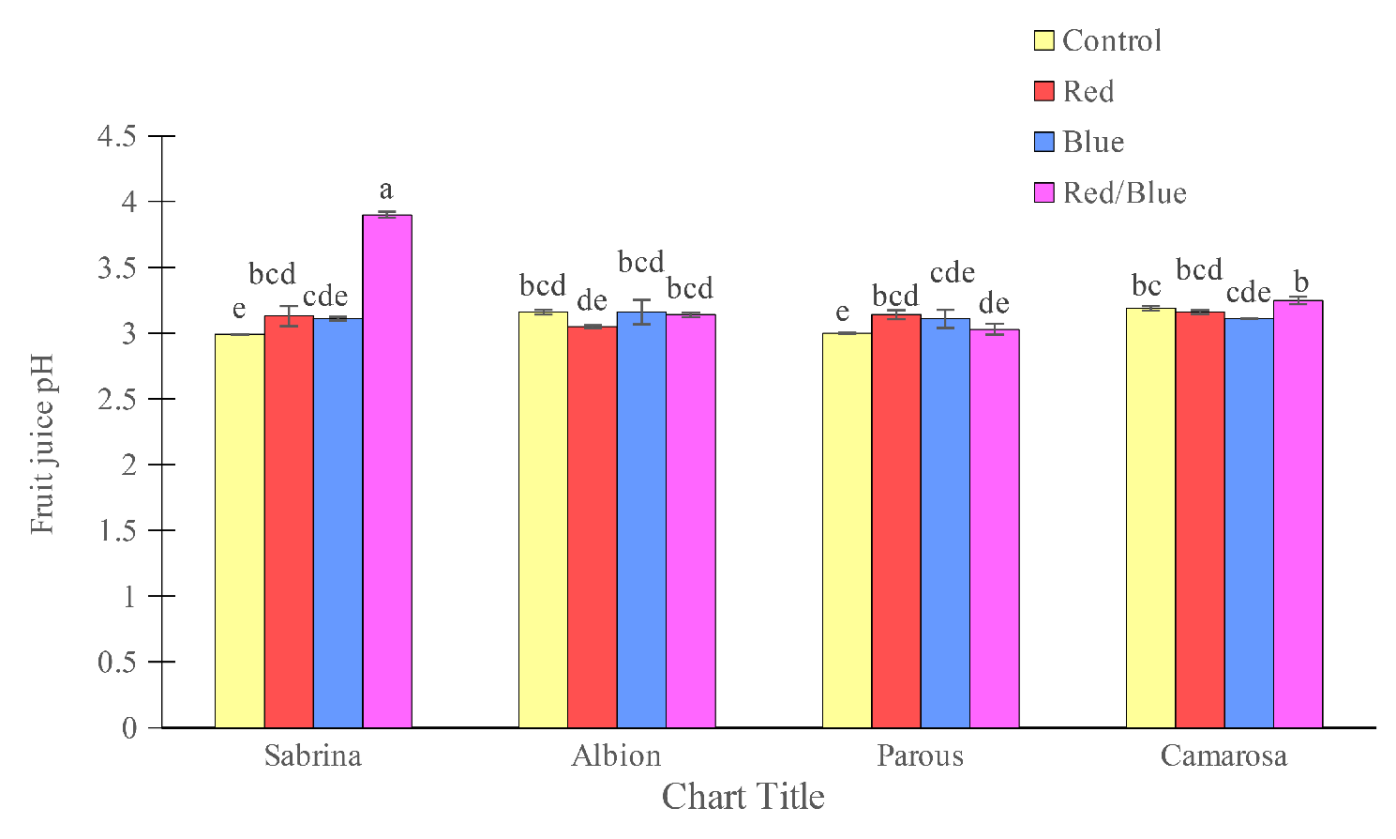
Effect of different supplemental LED light spectra (red/blue, 3:1; R: B, with a peak of 656 nm, blue, with a peak at 450 nm, red, with a peak at 656 nm, control (ambient light)) on fruit juice pH of four strawberry cultivars. The photosynthetic photon flux density (PPFD) was 215 ± 5 µmol m^− 2^ s^− 1^. The photoperiod of 11/13 h (day/ night) was maintained. Bars with different letters show significant differences at *P* ≤ 0.05 (Duncan)

**Fig. 9:**
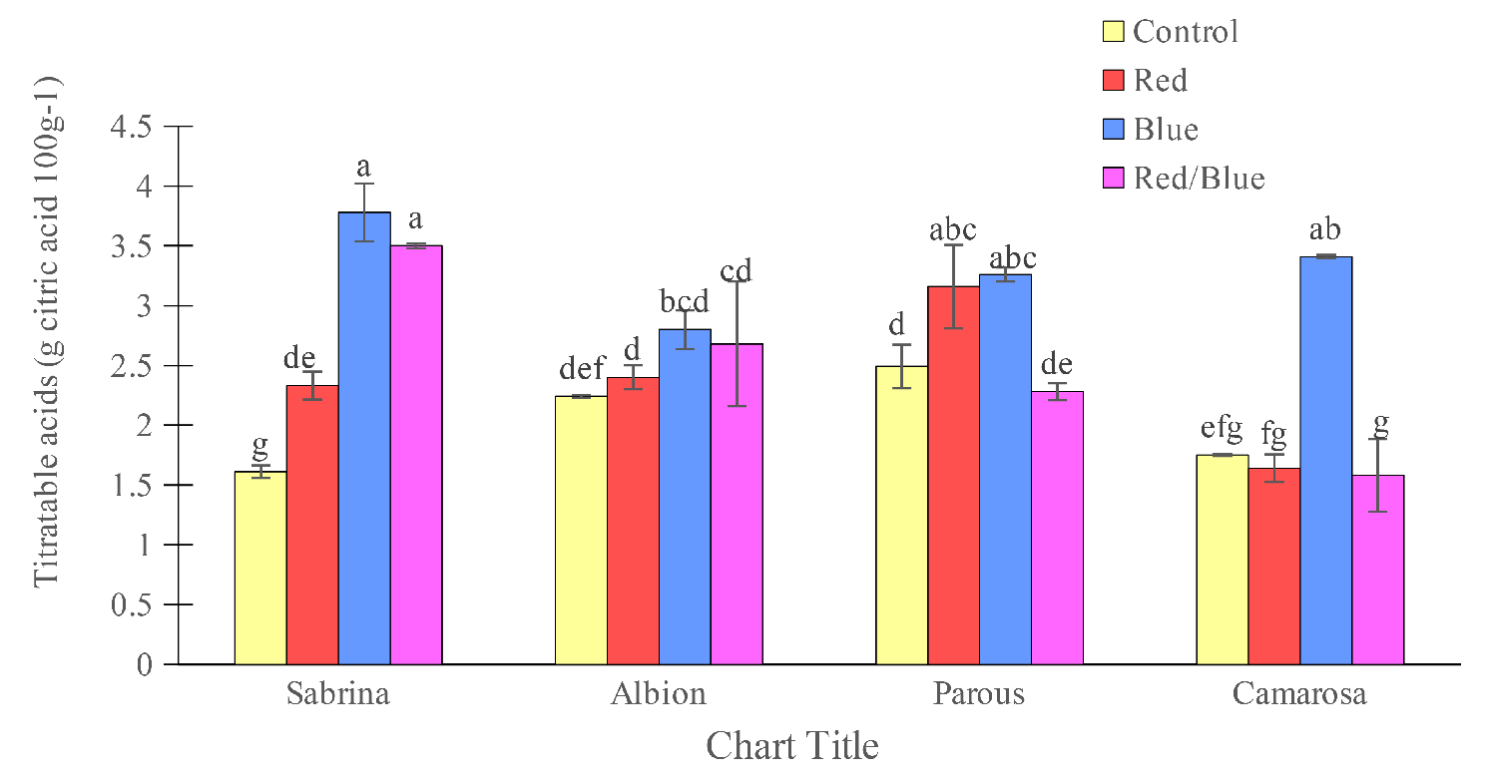
Effect of different supplemental LED light spectra (red/blue, 3:1; R: B, with a peak of 656 nm, blue, with a peak at 450 nm, red, with a peak at 656 nm, control (ambient light)) on titratable acids of four strawberry cultivars. The photosynthetic photon flux density (PPFD) was 215 ± 5 µmol m^− 2^ s^− 1^. The photoperiod of 11/13 h (day/ night) was maintained. Bars with different letters show significant differences at *P* ≤ 0.05 (Duncan)

The amount of total soluble solids in the fruit was found to be highest in the “Albion” and lowest in the “Parous” in the absence of supplemental light spectrums (Fig. 6). However, the use of supplemental light spectra significantly increased the fruit solids. Specifically, the use of blue light alone in “Sabrina” and “Camarosa”, blue and red/blue spectra in the “Parous”, and blue, red, and red/blue light spectra in the “Albion” increased the amount of soluble solids in the fruits. The anthocyanin concentration of the fruits was also found to be affected by the variety and light spectrum (Fig. 7). The “Albion” had the highest concentration of fruit anthocyanin, while the “Sabrina” had the lowest in the absence of supplemental light. However, the use of supplemental light spectra significantly increased the fruit anthocyanin concentration in all cultivars. The blue light spectrum was found to be the most effective in increasing the amount of anthocyanin in the fruit, especially in the “Camarosa”. The pH of the fruit juice was found to be highest in “Albion” and “Camarosa” and lowest in “Parous” and “Sabrina” in the absence of supplemental light (Fig. 8). However, the use of single red and red/blue light spectrums in “Sabrina” and red light spectrum in “Parous” increased the pH of the fruit juice. Lastly, titratable fruit acids was also found to be affected by cultivar and light spectrum. All the supplemental light spectra increased the titratable fruit acids in “Sabrina”, although, in “Parous” the red and blue, and in “Camarosa” only blue light increased the titratable fruit acids (Fig. 9).

### Elemental analysis

**Fig. 10:**
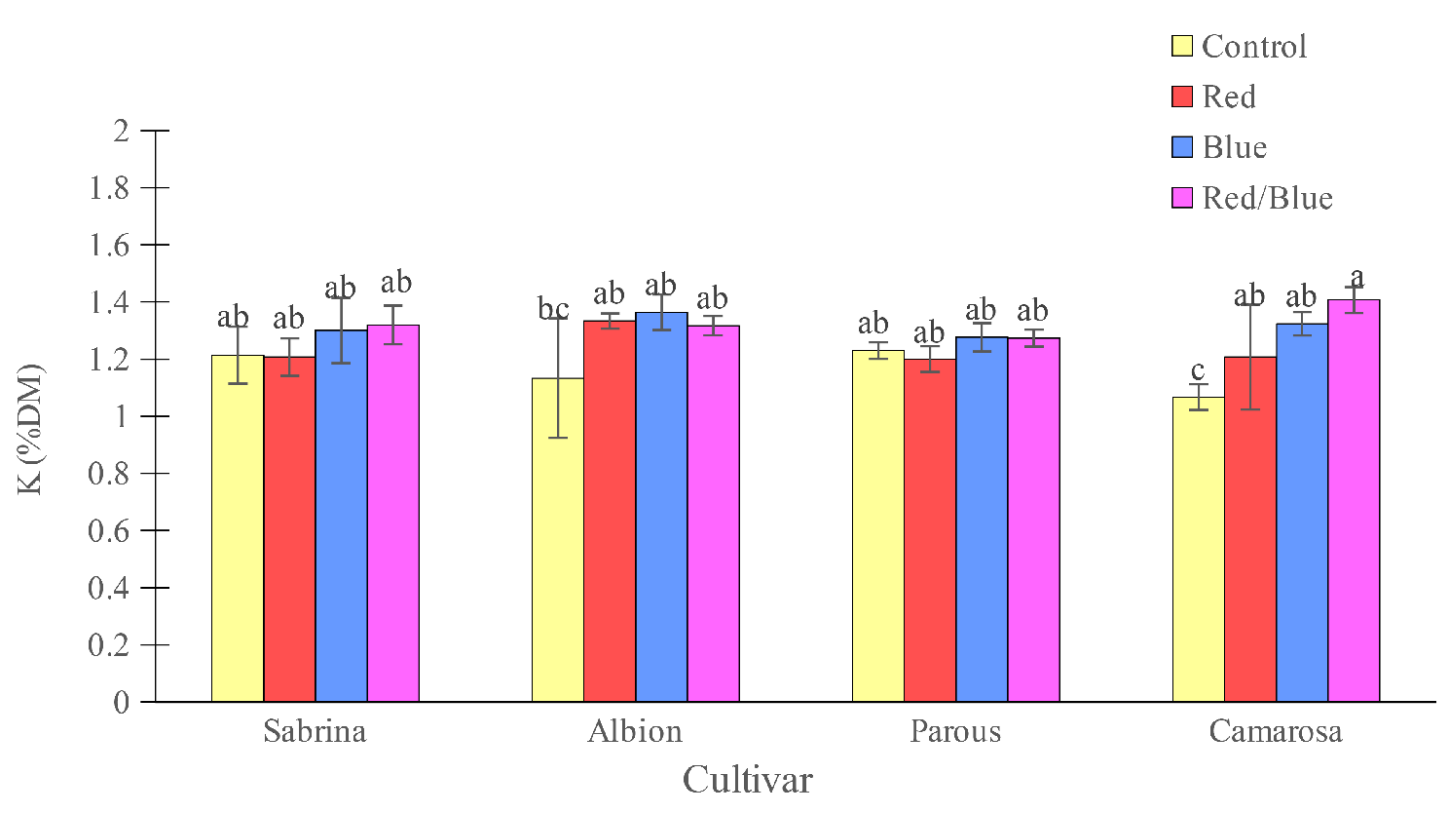
Effect of different supplemental LED light spectra (red/blue, 3:1; R: B, with a peak of 656 nm, blue, with a peak at 450 nm, red, with a peak at 656 nm, control (ambient light)) on leaf K concentration of four strawberry cultivars. The photosynthetic photon flux density (PPFD) was 215 ± 5 µmol m^− 2^ s^− 1^. The photoperiod of 11/13 h (day/ night) was maintained. Bars with different letters show significant differences at *P* ≤ 0.05 (Duncan)

**Fig. 11:**
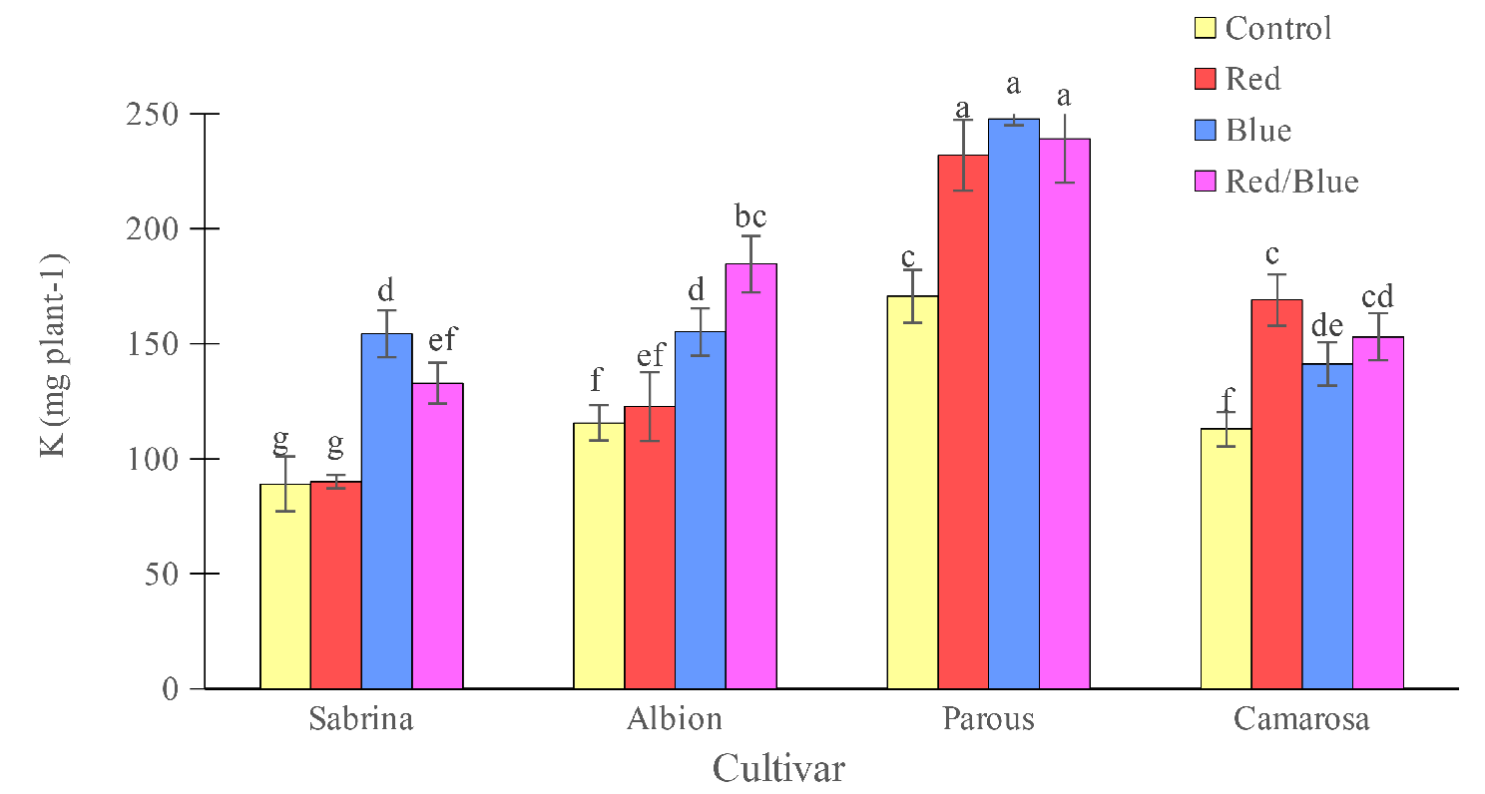
Effect of different supplemental LED light spectra (red/blue, 3:1; R: B, with a peak of 656 nm, blue, with a peak at 450 nm, red, with a peak at 656 nm, control (ambient light)) on leaf Mg concentration of four strawberry cultivars. The photosynthetic photon flux density (PPFD) was 215 ± 5 µmol m^− 2^ s^− 1^. The photoperiod of 11/13 h (day/ night) was maintained. Bars with different letters show significant differences at *P* ≤ 0.05 (Duncan)

**Fig. 12:**
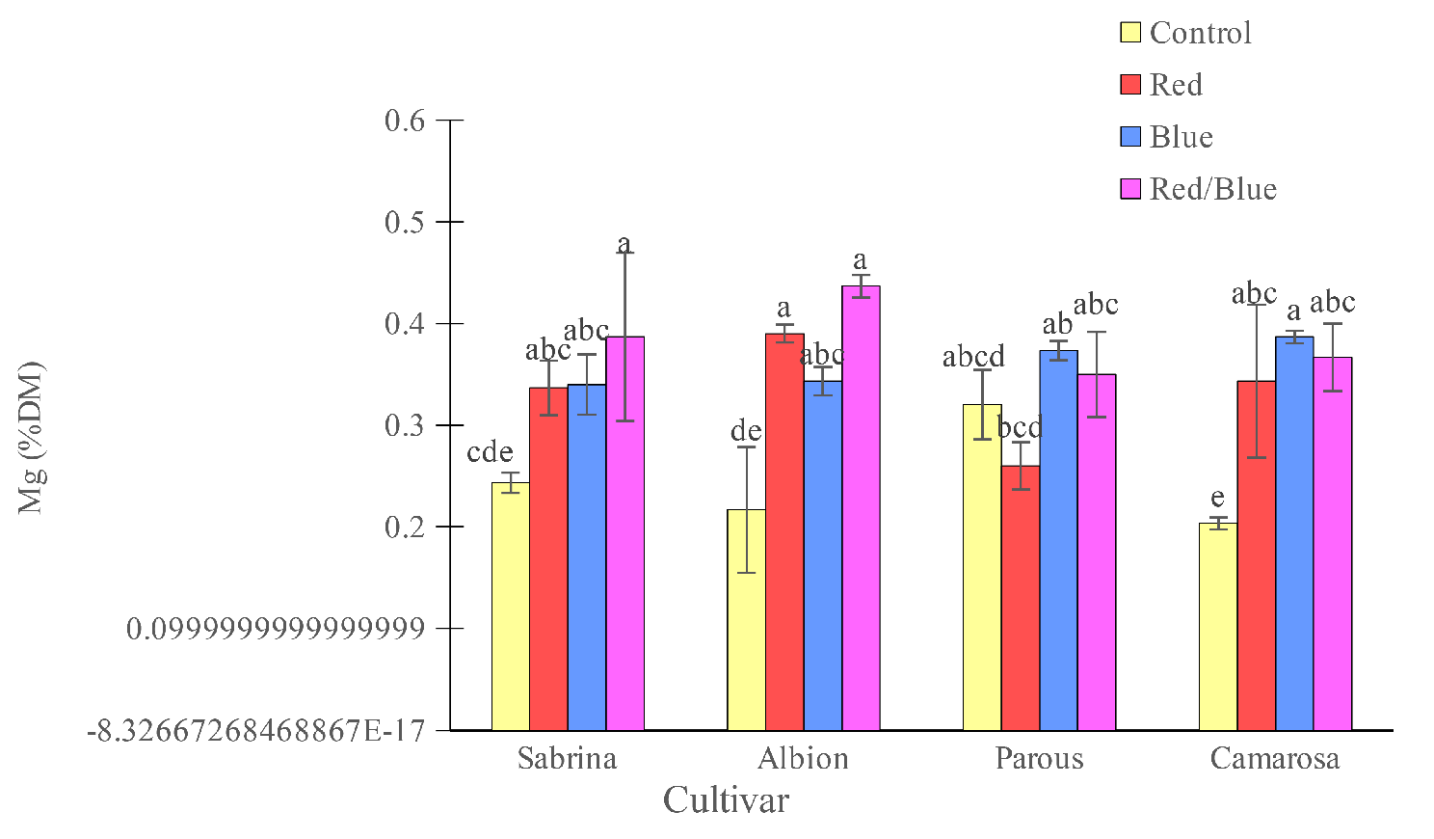
Effect of different supplemental LED light spectra (red/blue, 3:1; R: B, with a peak of 656 nm, blue, with a peak at 450 nm, red, with a peak at 656 nm, control (ambient light)) on K accumulation in four strawberry cultivars. The photosynthetic photon flux density (PPFD) was 215 ± 5 µmol m^− 2^ s^− 1^. The photoperiod of 11/13 h (day/ night) was maintained. Bars with different letters show significant differences at *P* ≤ 0.05 (Duncan)

**Fig. 13:**
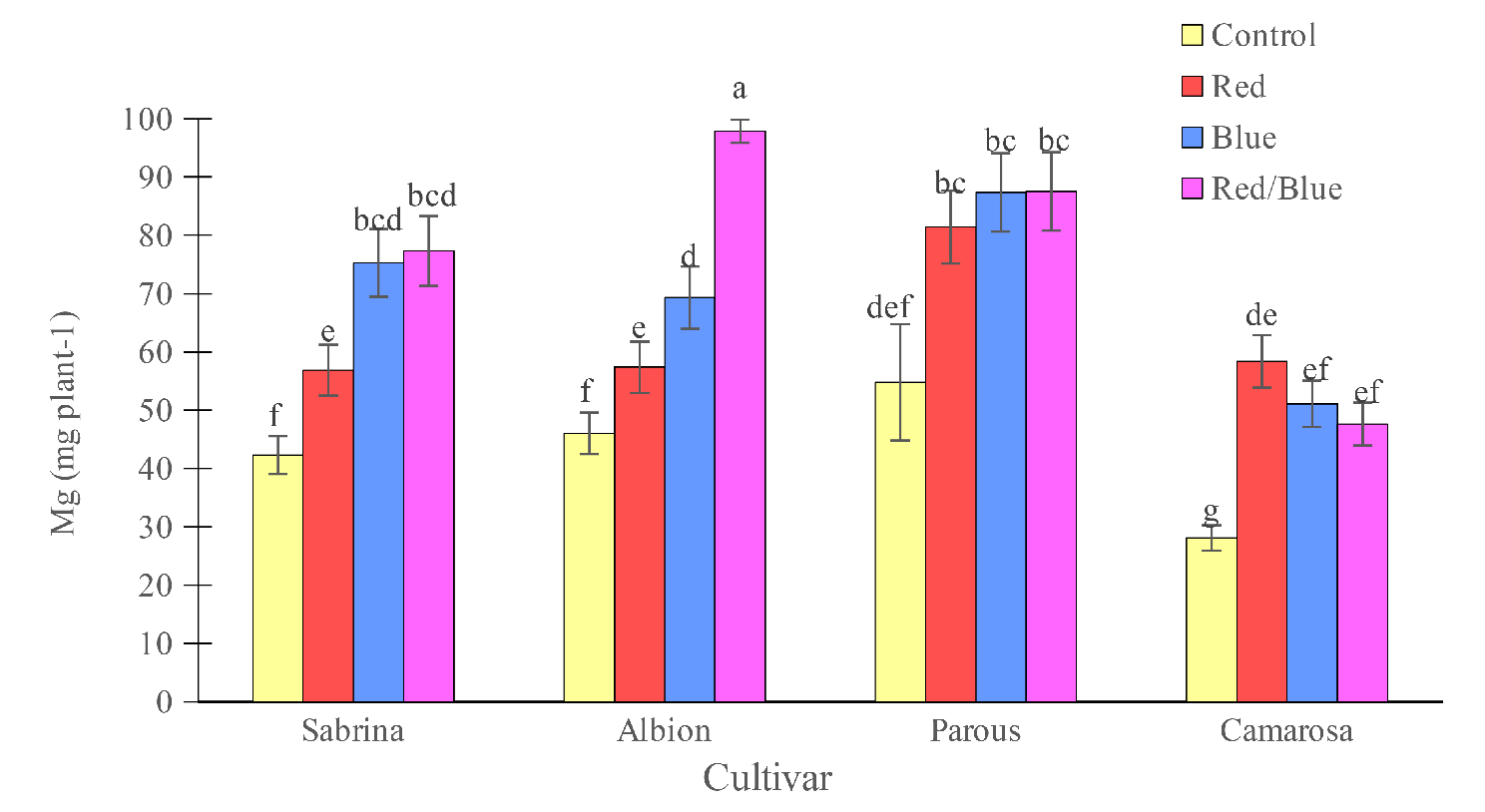
Effect of different supplemental LED light spectra (red/blue, 3:1; R: B, with a peak of 656 nm, blue, with a peak at 450 nm, red, with a peak at 656 nm, control (ambient light)) on Mg accumulation in four strawberry cultivars. The photosynthetic photon flux density (PPFD) was 215 ± 5 µmol m^− 2^ s^− 1^. The photoperiod of 11/13 h (day/ night) was maintained. Bars with different letters show significant differences at *P* ≤ 0.05 (Duncan)

The “Camarosa” had the lowest concentration of K and Mg when grown without supplemental light. However, the use of supplemental light spectrums showed an increase in both K and Mg concentrations (Figs. 10 and 11). For other cultivars, the effect of supplemental light treatment was not significant on K concentration, but Mg concentration increased significantly in the “Sabrina” with red/blue light spectrum treatment and in the “Albion” with all three applied spectrums. Magnesium concentration in the “Parous” was not affected by supplemental light spectrums. The results showed that, only blue and red/blue lights increased the accumulation of K in the “Sabrina” and “Albion” (Fig. 12). However, in the case of “Parous” and “Camarosa”, all supplemental light spectra led to an increase in K accumulation. Additionally, it was found that all supplemental lights increased Mg accumulation in all the cultivars that were studied (Fig. 13).

**Fig. 14:**
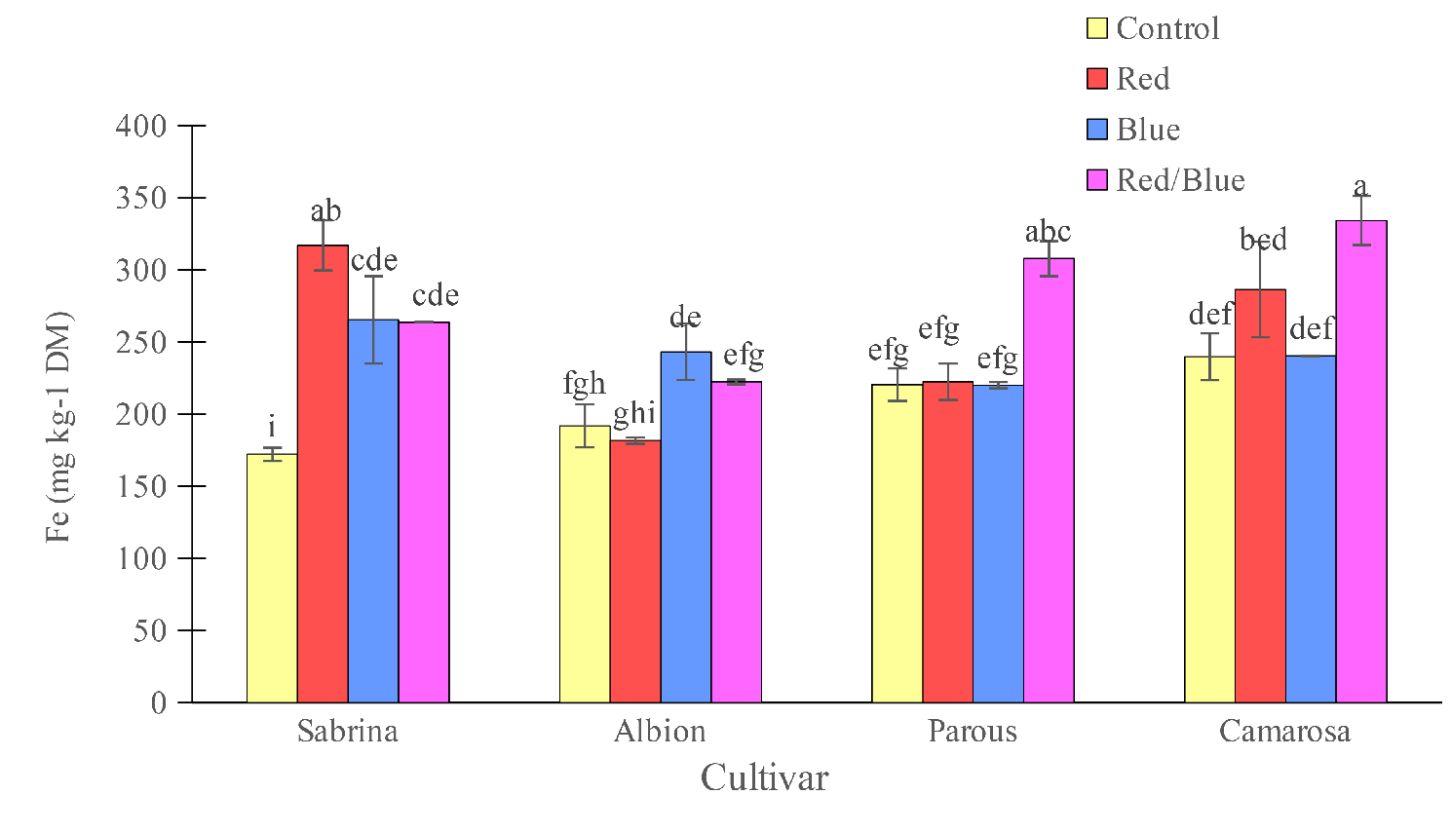
Effect of different supplemental LED light spectra (red/blue, 3:1; R: B, with a peak of 656 nm, blue, with a peak at 450 nm, red, with a peak at 656 nm, control (ambient light)) on leaf Fe concentration of four strawberry cultivars. The photosynthetic photon flux density (PPFD) was 215 ± 5 µmol m^− 2^ s^− 1^. The photoperiod of 11/13 h (day/ night) was maintained. Bars with different letters show significant differences at *P* ≤ 0.05 (Duncan)

**Fig. 15:**
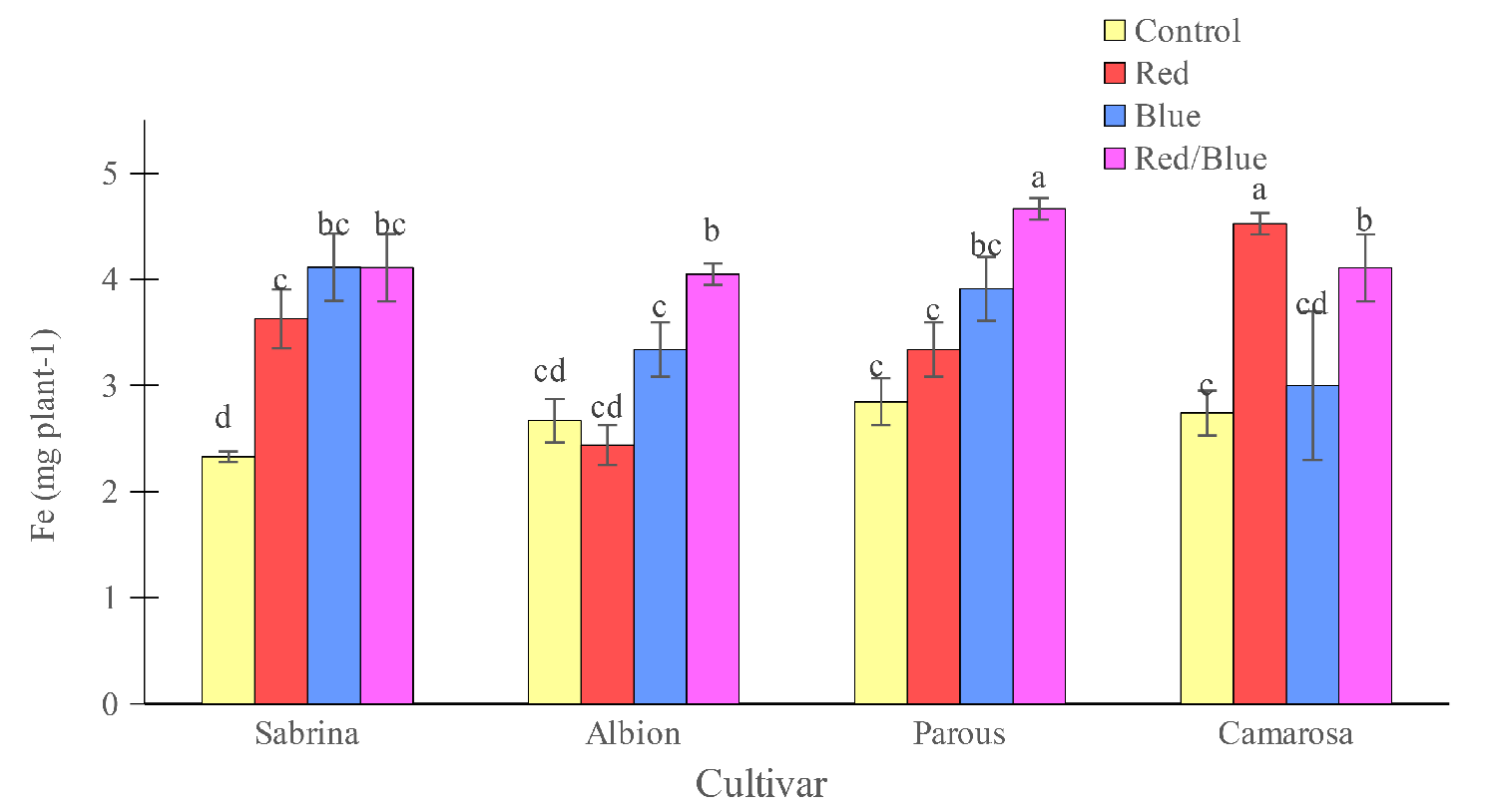
Effect of different supplemental LED light spectra (red/blue, 3:1; R: B, with a peak of 656 nm, blue, with a peak at 450 nm, red, with a peak at 656 nm, control (ambient light)) on Fe accumulation in four strawberry cultivars. The photosynthetic photon flux density (PPFD) was 215 ± 5 µmol m^− 2^ s^− 1^. The photoperiod of 11/13 h (day/ night) was maintained. Bars with different letters show significant differences at *P* ≤ 0.05 (Duncan)

All three spectrums tested increased the Fe concentration to the highest value in the “Sabrina”, although only red/blue light spectrum treatment was significant in the “Camarosa” (Fig. 14). In the “Albion”, only blue light spectrum, and in “Parous” only red/blue light spectrum increased the Fe concentration of leaves. In “Sabrina”, all supplemental light spectra increased Fe accumulation compared to the control, while in the other three cultivars red/blue supplemental light increased Fe accumulation (Fig. 15). In “Camarosa”, red light resulted in the highest Fe accumulation.

## Discussion

Light is crucial for plant growth and development [21]. Plants rely on light to complete their lifecycle, and the effects of different light spectra on plant growth vary [22, 23]. By adjusting the wavelength of light, seed germination, photosynthesis, biomass accumulation, and stomatal opening and closing can be optimized [24]. Photoreceptors allow plants to sense changes in light quality and regulate growth and development through signaling pathways. The intensity and quality of light significantly impact the morphology, physiology, and nutritional quality of plants [25]. Furthermore, light spectra affect plant growth, development, and morphogenesis [26]. Blue light is particularly important for chlorophyll biosynthesis and stomata closure. Recent studies have shown that blue supplemental LED light increases the concentration of total chlorophyll in “Albion” and “Camarosa”. A combination of blue and red light spectra is also crucial for leaf area and plant biomass production [15]. Other studies have observed that the red/blue light combination can increase the vegetative and reproductive traits of plants in greenhouse conditions [27]. For instance, under red and red/blue light, lettuce has a greater leaf area [15].

Studies have shown that red/blue light may increase the shoot dry mass of plants. This effect is caused by the impact of these light spectra on leaf area and leaf number [28]. The current experiments found that “Sabrina” and “Albion” exhibited a significant increase in root fresh mass under red/blue light, although root length and mass remained unaffected. Red light can stimulate phytochromes, leading to increased cell division and expansion, hypocotyl growth, and stem length [29, 30]. The combination of red and blue light has been found to promote more plant growth than monochromatic light [31]. This was observed in coriander plants, where fresh weight, dry weight, stem length, and leaf area were all higher when the proportion of red light was high [32], as well as in tomato [33] and cannabis [34]. However, some studies have found that plants exposed to high ratios of blue light exhibit weaker growth. This has been observed in wheat [35] and sage [36]. The results of current experiment showed that depending on the cultivar, blue, red, and red/blue lights can significantly increase vegetative growth, as evidenced by higher dry biomass of the shoot, root, and crown.

The current experiment findings provide evidence of an increased flower production per plant by supplemental lights in research greenhouses. These results agree with the findings of Díaz-Galián et al. [37] and Yoshida et al. [38] in greenhouse strawberry, and contradict previous reports from growth chambers, such as the study conducted by Nadalini et al. [39], where the number of flowers remained unchanged. Furthermore, another study conducted by Hyo et al. [40] demonstrated that the use of red/blue light combinations resulted in higher fruit yields compared to using single wavelengths. The introduction of supplemental light in strawberry cultivation stimulates photosynthesis, resulting in an increased supply of carbohydrates. This surplus of carbohydrates benefits the leaf primordia, leading to enhanced growth and ultimately contributing to a higher strawberry yield [41]. The quality of light is also shown to influence fruit size. A study conducted by Díaz-Galián et al. [37] found that a combination of blue and red light remarkably increases strawberry fruit production. Similarly, Hidaka et al. [5] discovered that red and blue light can lead to a significant increase in harvested fruits, while also shortening the period of fruit maturity. For strawberry plants (*Fragaria × ananassa* Duch), optimal growth and larger fruit production require a combination of red and blue spectra. This is because a high amount of glucose is produced in the leaf and transferred to the fruit under LED irradiation, resulting in an increase in average fruit mass. In the current experiment, the “Parous” showed no significant effect due to any of the supplemental light spectrums and exhibited the highest early fruit yield in control condition. However, for three other cultivars, all three tested supplemental light spectrums caused a significant increase in early fruit yield, with the “Albion” achieving the highest level. In the absence of supplemental light, the fruit mass and fruit length of the cultivars did not differ significantly. However, with the use of a red/blue supplemental light spectrum, these two traits increased significantly in the “Albion”. Therefore, the results of current research support the notion that the implementation of specific supplemental light spectra, can positively impact flower production in research greenhouses and improve fruit yields in controlled environments such as plastic greenhouses.

The chloroplasts in plants mainly use blue and red light for photosynthesis [42]. During photosynthesis, photosynthetic pigments play a vital role in absorbing and transferring light energy [43]. Studies have shown that blue and red light treatments can enhance the expression of genes that encode enzymes related to chlorophyll and carotenoid pigments [44]. These results in increased pigment accumulation. The combination of blue and red light has been found to be especially effective in increasing the concentration of chlorophyll [45]. The findings of current experiment suggest that the photosynthetic response of strawberry cultivars to supplemental light spectra varies. “Sabrina” responded best to red/blue light, while “Albion” did not show any significant differences among the supplemental light treatments. In “Camarosa” and “Parous,” red light was found to have the greatest positive impact on the rate of CO_2_ assimilation. These results demonstrate the importance of considering light spectra in optimizing photosynthetic activity in different strawberry cultivars. The results of current experiment highlights the variations in stomatal conductance response to different supplemental light spectra treatments among different strawberry varieties. It emphasizes the positive effect of red light on stomatal conductance in “Parous,” “Albion,” “Sabrina,” and “Camarosa” varieties, while blue light had a negative effect on stomatal conductance in the “Parous” variety specifically. Yanagi et al. [46] demonstrated that strawberry plants grown under red LEDs exhibited a higher rate of photosynthesis compared to those grown under blue LEDs.

Díaz-Galián et al. [37] found no significant differences in Brix grades of strawberry fruits between treatments using different light spectra in commercial greenhouses. Despite of this report, the current study demonstrated that the use of supplemental complementary light spectra can enhance the amount of soluble solids in different fruit cultivars, with specific combinations of light spectra yielding the best results for each cultivar. Plant secondary metabolites, such as phenolic compounds, have an important role in light signaling and protecting plants against environmental biotic and abiotic stresses [47]. Phenolic compounds, which contain at least one phenol functional group in their structure, are subdivided into several types, including phenolic acids, quinones, flavonoids, coumarins, and tannins [48]. Anthocyanin, a type of flavonoid, is synthesized through the phenylpropanoid pathway and is one of the most common secondary metabolites found in strawberry fruits. This study demonstrates that exposure to red and blue light, either alone or in combination, increases the concentration of anthocyanin in strawberry fruits. Light is a significant environmental factor that affects anthocyanin accumulation, and the quality of light is particularly important. Blue and red light have been found to induce anthocyanin biosynthesis when compared to darkness [49]. The impacts of light intensity level and spectrum on anthocyanin accumulation are attributed to their effect on expression of genes involved in anthocyanin biosynthesis [50]. The molecular relationship between blue light receptors and anthocyanin accumulation has been extensively studied. High expression of phototropin has been found to correspond to increased anthocyanin content [51]. Additionally, blue light irradiation led to overexpression of cryptochrome, resulting in increase of anthocyanin accumulation [52]. Blue light is absorbed by cryptochromes, which affect different phases of plant growth, including the production of secondary fruit metabolites [53]. The biosynthesis of phenolic and anthocyanin compounds with blue light has also been demonstrated in tomatoes [54] and grapes [55]. The effect of supplemental blue and red light on anthocyanin accumulation has also been extensively investigated in many greenhouse plants and fruit crops [56].

Plants require a number of essential nutrients, one of which is potassium. This mineral is highly concentrated in the cytoplasm of plant cells, ranging from 100 to 200 mM. It plays a crucial role in regulating plant growth and metabolism and affects most of the plant’s biochemical and physiological processes including osmotic regulation, enzyme activation, stomatal regulation, protein synthesis, carbohydrate metabolism, ion balance [57]. Another essential element for plant growth and reproduction is magnesium, which is used in significant amounts. Magnesium is responsible for various physiological functions in plant cells, including serving as a central atom in chlorophyll, a vital component for photosynthesis [58]. It is also involved in CO_2_ absorption reactions that occur in the chloroplast [59]. Iron is also a vital component for plant growth, and it is essential for photosynthesis and chlorophyll synthesis. It’s a crucial component of electron chains and many vital enzymes in plants. The availability of iron in the soil determines the distribution of plant species in natural ecosystems and limits the yield and nutritional quality of crops. Insufficient absorption of iron leads to delayed growth, chlorosis between the veins, and reduced plant growth. Therefore, plants must use strategies to increase their mobility and absorb it sufficiently [60]. Mineral content in plants depends on several factors, including species and variety, maturity stages, growing seasons, and environmental factors such as light during the growth period [61]. light is crucial for nutrient uptake in plants because it provides the energy for photosynthesis, regulates stomatal opening for gas and water exchange, influences hormonal responses, and affects root growth and development [3]. By optimizing light conditions, such as intensity, duration, and quality, it is possible to enhance nutrient uptake and overall plant productivity. It is established that light quality can trigger metabolic modifications in plants [62]. The current research has proven that the combination of blue and red light, which creates a supplemental light spectrum, leads to a higher mineral content in strawberry plants. Blue light plays a crucial role in this process as it is controlled by the phototropin receptor and opens ion channels found in the cell plasma membrane, which increases the ion transport current [63]. Blue light also plays a crucial role in the signaling process of cryptochromes, which are blue-light receptors that utilize blue-light energy to enhance the uptake of macro and micronutrients [64]. Several studies have shown that blue light supplementation has a positive effect on the accumulation of minerals in different types of plants [65]. Similarly, research has also indicated that red light has a beneficial effect on the mineral content of beet [66]. Red LED lights are believed to affect metabolic pathways and water absorption, leading to an increase in mineral element contents in leaves [67]. Limited information is currently available on the effects of blue and red light spectrums on the mineral content in strawberry cultivars. However, studies have been conducted on different ratios of blue and red light, which have shown similar trends in mineral nutrients in plants [68]. Research has revealed that the combination of 25% blue light and red light increases the mineral content in mustard plants [69]. There is not much information available on how mineral nutrients in plants are affected by varying light levels, according to a study by Alrifai et al. [70]. There are conflicting reports on the impact of red/blue light and monochromatic blue-red light with different ratios on mineral content in different plants, which depends on genotype and minerals. For instance, some researchers have found that the mineral content in marigolds is not significantly different among treatments with different percentages of blue light, as per a study conducted by Sams et al. [71]. However, the combination of blue and red light with a significant percentage of red light has been found to increase the content of certain minerals such as P, K, Ca, and Zn in plants [72], as well as N, P, K, and Mg in lettuce [73] and various minerals in basil [74]. Additionally, previous studies have shown a positive effect of red light on mineral content in various plants. For example, research has demonstrated that red light plays a crucial role in the absorption of mineral nutrients and the stimulation of roots through multiple pathways, thanks to phytochrome photoreceptors in Arabidopsis [75]. On the other hand, a higher percentage of blue light leads to an increase in Mg in basil [76] and Ca in lettuce [67]. While studies have shown how different blue-to-red LED light ratios affect the uptake of mineral nutrients in aerial organs, there is limited information available on their impact on the uptake of minerals from the hydroponic nutrient solution into roots and from roots to shoots.

There are reports that suggest younger plants are more capable of absorbing metal ions [77]. This could be due to their more intense transpiration during leaf enlargement and stomatal development [78]. Additionally, some research indicates that metal elements can be transported to shoots as non-toxic elements, thanks to metal sequestration in plant root vacuoles [66]. Blue-to-red light ratios also play a role in mineral nutrient translocation in microgreens, but different plant species respond differently to different combinations of blue and red light, which affects their mineral nutrient uptake [79]. Similarly, the present study highlights the varying effects of different supplemental light spectra on K and Fe accumulation among different strawberry cultivars, while also demonstrating a consistent increase in Mg accumulation across all cultivars.

## Conclusions

Different cultivars of strawberries have distinct growth strategies, which are influenced by the quality of light. A recent study examined how blue, red, and combined red-blue light affect the absorption of elements and biochemical traits of plants. Although LED technology shows promise for greenhouse cultivation, more research is needed to understand its effects on different plant varieties under varying environmental conditions. Appropriate light quality and intensity are critical to plant growth, and the optimal combination differs based on the species or variety. It can be concluded that light quality is a significant factor in strawberry plant growth, physiology and nutrition. Thus, providing a specific amount and quality of light is essential to achieve optimal growth for a particular species or variety.

## Data Availability

All the data generated or analyzed during the current study were included in the manuscript. The raw data is available from the corresponding author on reasonable request.

## Declarations

### Statement of compliance

The authors confirm that all the experimental research and field studies on strawberry plants, including the collection of plant material, complied with relevant institutional, national, and international guidelines and legislation. Also, obtained licenses for the preparation of Bare root plants of strawberry.

### Statement on experimental research and field studies on plants

The indoor-growing plants sampled comply with relevant institutional, national, and international guidelines and domestic legislation of Iran.

### Statement specifying permissions

In this study, we obtained permission to cultivate strawberry cultivars if issued by the Agricultural and Natural Resources Ministry of Iran.

## References

[1] Kozai T, Fujiwara K, Runkle ES. LED lighting for urban agriculture. LED Light Urban Agric. 2016;:1–454.

[2] Patil GG, Oi R, Gissinger A, Moe R (2001). Plant morphology is affected by light quality selective plastic films and alternating day and night temperature. Gartenbauwissenschaft.

[3] Malekzadeh Shamsabad MR, Esmaeilizadeh M, Roosta HR, Dąbrowski P, Telesiński A, Kalaji HM. Supplemental light application can improve the growth and development of strawberry plants under salinity and alkalinity stress conditions. Sci Rep. 2022;12. 10.1038/s41598-022-12925-8 PMC9166788 35661116

[4] Soufi HR, Roosta HR, Stępień P, Malekzadeh K, Hamidpour M. Manipulation of light spectrum is an effective tool to regulate biochemical traits and gene expression in lettuce under different replacement methods of nutrient solution. Sci Rep. 2023;13. 10.1038/s41598-023-35326-x PMC10219983 37237093

[5] Hidaka K, Dan K, Miyoshi Y, Imamura H, Takayama T, Kitano M (2016). Twofold increase in strawberry productivity by integration of environmental control and movable beds in a large-scale greenhouse. Environ Control Biol.

[6] Malekzadeh Shamsabad MR, Roosta HR, Esmaeilizadeh M (2021). Responses of seven strawberry cultivars to alkalinity stress under soilless culture system. J Plant Nutr.

[7] Hernández R, Kubota C (2016). Physiological responses of cucumber seedlings under different blue and red photon flux ratios using LEDs. Environ Exp Bot.

[8] Son K-H, Kim E-Y, Oh M-M (2018). Growth and development of Cherry Tomato seedlings grown under various combined ratios of red to blue LED lights and Fruit Yield and Quality after transplanting. Prot Hortic Plant Fact.

[9] Rahman MM, Field DL, Ahmed SM, Hasan MT, Basher MK, Alameh K. LED illumination for high-quality high-yield crop growth in protected cropping environments. Plants. 2021;10. 10.3390/plants10112470 PMC8621602 34834833

[10] Magar YG, Ohyama K, Noguchi A, Amaki W, Furufuji S (2018). Effects of light quality during supplemental lighting on the flowering in an everbearing strawberry. Acta Hortic.

[11] Samuoliene G, Brazaityte A, Urbonavičiute A, Šabajeviene G, Duchovskis P (2010). The effect of red and blue light component on the growth and development of frigo strawberries. Zemdirbyste.

[12] Liu XY, Chang TT, Guo SR, Xu ZG, Li J (2011). Effect of different light quality of LED on growth and photosynthetic character in cherry tomato seedling. Acta Hortic.

[13] Schuerger AC, Brown CS, Stryjewski EC. Anatomical features of pepper plants (Capsicum annum L.) grown under red light-emitting diodes supplemented with blue or far-red light. Ann Bot. 1997;79. 10.1006/anbo.1996.0341 11540425

[14] Takeda F, Glenn DM, Stutte GW (2008). Red light affects flowering under long days in a short-day strawberry cultivar. HortScience.

[15] Johkan M, Shoji K, Goto F, Hashida S, nosuke, Yoshihara T (2010). Blue light-emitting diode light irradiation of seedlings improves seedling quality and growth after transplanting in red leaf lettuce. HortScience.

[16] Muneer S, Kim EJ, Park JS, Lee JH (2014). Influence of green, red and blue light emitting diodes on multiprotein complex proteins and photosynthetic activity under different light intensities in lettuce leaves (Lactuca sativa L). Int J Mol Sci.

[17] Morgan L (2006). Hydroponic strawberry production. Grow Edge.

[18] Lichtenthaler HK (1987). Chlorophylls and carotenoids: pigments of photosynthetic biomembranes. Methods Enzymol.

[19] Nogues S (2000). Effects of drought on photosynthesis in Mediterranean plants grown under enhanced UV-B radiation. J Exp Bot.

[20] Amiri A, Mortazavi SMH, Ramezanian A, Mahmoodi Sourestani M, Mottaghipisheh J, Iriti M (2021). Prevention of decay and maintenance of bioactive compounds in strawberry by application of UV-C and essential oils. J Food Meas Charact.

[21] Kim TH, Kim BH, Von Arnim AG (2002). Repressors of photomorphogenesis. Int Rev Cytol.

[22] Ye S, Shao Q, Xu M, Li S, Wu M, Tan X et al. Effects of light quality on morphology, enzyme activities, and bioactive compound contents in Anoectochilus Roxburghii. Front Plant Sci. 2017;8. 10.3389/fpls.2017.00857 PMC5440764 28588604

[23] Fukuda N, Fujita M, Ohta Y, Sase S, Nishimura S, Ezura H (2008). Directional blue light irradiation triggers epidermal cell elongation of abaxial side resulting in inhibition of leaf epinasty in geranium under red light condition. Sci Hortic (Amsterdam).

[24] Saleem MH, Rehman M, Fahad S, Tung SA, Iqbal N, Hassan A (2020). Leaf gas exchange, oxidative stress, and physiological attributes of rapeseed (Brassica napus L.) grown under different light-emitting diodes. Photosynthetica.

[25] Ward JM, Cufr CA, Denzel MA, Neff MM (2005). The dof transcription factor OBP3 modulates phytochrome and cryptochrome signaling in arabidopsis. Plant Cell.

[26] Lanoue J, Leonardos ED, Grodzinski B. Effects of light quality and intensity on diurnal patterns and rates of photo-assimilate translocation and transpiration in tomato leaves. Front Plant Sci. 2018;9. 10.3389/fpls.2018.00756 PMC5994434 29915612

[27] Naznin MT, Lefsrud M, Gravel V, Hao X (2016). Using different ratios of red and blue LEDs to improve the growth of strawberry plants. Acta Hortic.

[28] Wang J, Lu W, Tong Y, Yang Q. Leaf morphology, photosynthetic performance, chlorophyll fluorescence, stomatal development of lettuce (Lactuca sativa L.) exposed to different ratios of red light to blue light. Front Plant Sci. 2016;7. 10.3389/fpls.2016.00250 PMC4785143 27014285

[29] Anuchai J, Hsieh CH (2017). Effect of change in light quality on physiological transformation of in vitro phalaenopsis ‘fortune saltzman’ seedlings during the growth period. Hortic J.

[30] Neff MM, Fankhauser C, Chory J (2000). Eight: an indicator of time and place. Genes Dev.

[31] Li Y, Xin G, Liu C, Shi Q, Yang F, Wei M. Effects of red and blue light on leaf anatomy, CO2assimilation and the photosynthetic electron transport capacity of sweet pepper (Capsicum annuum L.) seedlings. BMC Plant Biol. 2020;20. 10.1186/s12870-020-02523-z PMC7336438 32631228

[32] Gao Q, Liao Q, Li Q, Yang Q, Wang F, Li J. Effects of LED Red and Blue Light Component on Growth and photosynthetic characteristics of Coriander in Plant Factory. Horticulturae. 2022;8.

[33] Nanya K, Ishigami Y, Hikosaka S, Goto E (2012). Effects of blue and red light on stem elongation and flowering of tomato seedlings. Acta Hortic.

[34] LIVADARIU O, RAICIU D, MAXIMILIAN C, CĂPITANU E (2019). Studies regarding treatments of LED-s emitted light on sprouting hemp (Cannabis sativa L). Rom Biotechnol Lett.

[35] Li J, Guo X, Zhang S, Zhang Y, Chen L, Zheng W et al. Effects of light quality on growth, nutritional characteristics, and antioxidant properties of winter wheat seedlings (Triticum aestivum L). Front Plant Sci. 2022;13. 10.3389/fpls.2022.978468 PMC9478206 36119584

[36] Zhang S, Ma J, Zou H, Zhang L, Li S, Wang Y. The combination of blue and red LED light improves growth and phenolic acid contents in Salvia miltiorrhiza Bunge. Ind Crops Prod. 2020;158.

[37] Díaz-Galián MV, Torres M, Sanchez-Pagán JD, Navarro PJ, Weiss J, Egea-Cortines M (2021). Enhancement of strawberry production and fruit quality by blue and red LED lights in research and commercial greenhouses. South Afr J Bot.

[38] Yoshida H, Mizuta D, Fukuda N, Hikosaka S, Goto E (2016). Effects of varying light quality from single-peak blue and red light-emitting diodes during nursery period on flowering,photosynthesis,growth,and fruit yield of everbearing strawberry. Plant Biotechnol.

[39] Nadalini S, Zucchi P, Andreotti C (2017). Effects of blue and red led lights on soilless cultivated strawberry growth performances and fruit quality. Eur J Hortic Sci.

[40] Hyo GC, Byoung YM, Nam JK (2015). Effects of LED light on the production of strawberry during cultivation in a plastic greenhouse and in a growth chamber. Sci Hortic (Amsterdam).

[41] Hidaka K, Dan K, Imamura H, Takayama T, Sameshima K, Okimura M (2015). Variety comparison of effect of supplemental lighting with LED on growth and yield in forcing culture of strawberry. Environ Control Biol.

[42] Buschmann C, Langsdorf G, Lichtenthaler HK (2001). Imaging of the blue, green, and red fluorescence emission of plants: an overview. Photosynthetica.

[43] Simkin AJ, Kapoor L, Doss CGP, Hofmann TA, Lawson T, Ramamoorthy S (2022). The role of photosynthesis related pigments in light harvesting, photoprotection and enhancement of photosynthetic yield in planta. Photosynth Res.

[44] Ma G, Zhang L, Kato M, Yamawaki K, Kiriiwa Y, Yahata M (2012). Effect of blue and red LED light irradiation on β-cryptoxanthin accumulation in the flavedo of citrus fruits. J Agric Food Chem.

[45] Huang B, Lin B, Li C, Liu X, Liao Z, Liu Y (2018). Effects of LED light quality on growth and photosynthetic physiological characteristics in spinach. J Fujian Agric Univ (Natural Sci Ed.

[46] Yanagi T, Okamoto K, Takita S (1996). Effect of blue and red light intensity on photo synthetic rate of strawberry leaves. Acta Hortic.

[47] Durairaj T, Alagappan C, Suresh SS, R, Ramasamy V. An introductory chapter: secondary metabolites. Second Metab - Sources Appl. 2018;:2–21.

[48] Li J, Huang Y, Chen L (2023). Understory plant diversity and phenolic allelochemicals across a range of Eucalyptus grandis plantation ages. J Res.

[49] Liu Z, Zhang Y, Wang J, Li P, Zhao C, Chen Y (2015). Phytochrome-interacting factors PIF4 and PIF5 negatively regulate anthocyanin biosynthesis under red light in Arabidopsis seedlings. Plant Sci.

[50] Liu Y, Schouten RE, Tikunov Y, Liu X, Visser RGF, Tan F et al. Blue light increases anthocyanin content and delays fruit ripening in purple pepper fruit. Postharvest Biol Technol. 2022;192.

[51] Kadomura-Ishikawa Y, Miyawaki K, Noji S, Takahashi A (2013). Phototropin 2 is involved in blue light-induced anthocyanin accumulation in Fragaria x ananassa fruits. J Plant Res.

[52] Giliberto L, Perrotta G, Pallara P, Weller JL, Fraser PD, Bramley PM (2005). Manipulation of the blue light photoreceptor cryptochrome 2 in tomato affects vegetative development, flowering time, and fruit antioxidant content. Plant Physiol.

[53] Fantini E, Facella P (2020). Cryptochromes in the field: how blue light influences crop development. Physiol Plant.

[54] Lopez L, Carbone F, Bianco L, Giuliano G, Facella P, Perrotta G (2012). Tomato plants overexpressing cryptochrome 2 reveal altered expression of energy and stress-related gene products in response to diurnal cues. Plant Cell Environ.

[55] González VerónicaC, Fanzone, Leandro M, Cortés Emanuel L, Bottini R, Lijavetzky, Claudio D, Ballaré, Luis C et al. Fruit-localized photoreceptors increase phenolic compounds in berry skins of field-grown Vitis vinifera L. cv. Malbec. Phytochemistry. 2015;110:46–57. 10.1016/j.phytochem.2014.11.018 25514818

[56] Liu Y, Tikunov Y, Schouten RE, Marcelis LFM, Visser RGF, Bovy A. Anthocyanin biosynthesis and degradation mechanisms in Solanaceous vegetables: A review. Front Chem. 2018;6 MAR. 10.3389/fchem.2018.00052 PMC5855062 29594099

[57] Wang M, Zheng Q, Shen Q, Guo S (2013). The critical role of potassium in plant stress response. Int J Mol Sci.

[58] Rissler HM (2002). Chlorophyll biosynthesis. Expression of a second chl I gene of Magnesium Chelatase in Arabidopsis supports only limited chlorophyll synthesis. Plant Physiol.

[59] Tränkner M, Tavakol E, Jákli B (2018). Functioning of potassium and magnesium in photosynthesis, photosynthate translocation and photoprotection. Physiol Plant.

[60] Schmidt W, Thomine S, Buckhout TJ. Editorial: Iron Nutrition and interactions in plants. Front Plant Sci. 2020;10. 10.3389/fpls.2019.01670 PMC6968163 31998349

[61] Kyriacou MC, Rouphael Y, Di Gioia F, Kyratzis A, Serio F, Renna M (2016). Micro-scale vegetable production and the rise of microgreens. Trends Food Sci Technol.

[62] Liu Y, Roof S, Ye Z, Barry C, Van Tuinent A, Vrebalov J (2004). Manipulation of light signal transduction as a means of modifying fruit nutritional quality in tomato. Proc Natl Acad Sci U S A.

[63] Xu J, Guo Z, Jiang X, Ahammed GJ, Zhou Y (2021). Light regulation of horticultural crop nutrient uptake and utilization. Hortic Plant J.

[64] Jing Y. Cryptochrome effect on mineral element absorption. Hunan University; 2009.

[65] Brazaitytė A, Vaštakaitė-Kairienė V, Jankauskienė J, Viršilė A, Samuolienė G, Sakalauskienė S (2020). Effect of blue light percentage on mineral elements content in Brassica microgreens. Acta Hortic.

[66] Brazaitytė A, Miliauskienė J, Vaštakaitė-Kairienė V, Sutulienė R, Laužikė K, Duchovskis P et al. Effect of different ratios of blue and red led light on brassicaceae microgreens under a controlled environment. Plants. 2021;10. 10.3390/plants10040801 PMC8073284 33921895

[67] Amoozgar A, Mohammadi A, Sabzalian MR (2017). Impact of light-emitting diode irradiation on photosynthesis, phytochemical composition and mineral element content of lettuce cv. Grizzly Photosynthetica.

[68] Bartucca ML, Del Buono D, Ballerini E, Benincasa P, Falcinelli B, Guiducci M. Effect of light spectrum on gas exchange, growth and biochemical characteristics of einkorn seedlings. Agronomy. 2020;10.

[69] Brazaitytė A, Vaštakaitė V, Viršilė A, Jankauskienė J, Samuolienė G, Sakalauskienė S (2018). Changes in mineral element content of microgreens cultivated under different lighting conditions in a greenhouse. Acta Hortic.

[70] Alrifai O, Hao X, Marcone MF, Tsao R (2019). Current review of the Modulatory effects of LED lights on photosynthesis of secondary metabolites and future perspectives of Microgreen vegetables. J Agric Food Chem.

[71] Sams CE, Kopsell D, Morrow RC (2016). Light quality impacts on growth, flowering, mineral uptake and petal pigmentation of marigold. Acta Hortic.

[72] Frąszczak B, Gąsecka M, Golcz A, Zawirska-Wojtasiak R (2016). The effect of radiation of LED modules on the growth of dill (Anethum graveolens L). Open Life Sci.

[73] Pennisi G, Orsini F, Blasioli S, Cellini A, Crepaldi A, Braschi I et al. Resource use efficiency of indoor lettuce (Lactuca sativa L.) cultivation as affected by red:blue ratio provided by LED lighting. Sci Rep. 2019;9. 10.1038/s41598-019-50783-z PMC6773742 31576006

[74] Kamal KY, Khodaeiaminjan M, El-Tantawy AA, Moneim DA, Salam AA, Ash-shormillesy SMAI (2020). Evaluation of growth and nutritional value of Brassica microgreens grown under red, blue and green LEDs combinations. Physiol Plant.

[75] Sakuraba Y, Yanagisawa S (2018). Light signalling-induced regulation of nutrient acquisition and utilisation in plants. Semin Cell Dev Biol.

[76] Hammock H (2018). The Impact of Blue and red LED lighting on Biomass Accumulation, Flavor Volatile Production, and nutrient uptake in hydroponically grown Genovese Basil. Masters Theses.

[77] Wang RH, Chang JC, Li KT, Lin TS, Chang LS (2014). Leaf age and light intensity affect gas exchange parameters and photosynthesis within the developing canopy of field net-house-grown papaya trees. Sci Hortic (Amsterdam).

[78] Li X, Wu Y, Li B, Yang Y, Yang Y. Selenium accumulation characteristics and biofortification potentiality in turnip (Brassica rapa var. rapa) supplied with selenite or selenate. Front Plant Sci. 2018;8. 10.3389/fpls.2017.02207 PMC5758583 29354147

[79] Clavijo-Herrera J, Van Santen E, Gómez C. Growth, water-use efficiency, stomatal conductance, and nitrogen uptake of two lettuce cultivars grown under different percentages of blue and red light. Horticulturae. 2018;4.

